# Bridging the evidence-to-practice gap in cardiometabolic prevention: from episodic care to lifelong, system-wide responsibility

**DOI:** 10.3389/fpubh.2026.1903747

**Published:** 2026-09-14

**Authors:** Wirginia Krzyściak, Paweł Latacz, Maciej Chwała, Anna Lizoń, Małgorzata Trofimiuk-Müldner

**Affiliations:** 1 Department of Medical Diagnostic, Faculty of Pharmacy, Jagiellonian University Medical College, Kraków, Poland; 2 Department of Vascular Surgery and Angiology, St. John Grande Hospital, Kraków, Poland; 3 Department of Radiology, Faculty of Medicine, Jagiellonian University Medical College, Kraków, Poland; 4 Clinic of Surgery, Institute of Physiotherapy, Jagiellonian University Medical College, Kraków, Poland; 5 Department of Endocrinology, Faculty of Medicine, Jagiellonian University Medical College, Kraków, Poland

**Keywords:** apolipoprotein B, cardiometabolic diseases, cardiovascular risk, healthcare systems, interdisciplinary care, lipoprotein(a), metabolic continuum, prevention

## Abstract

Cardiometabolic diseases—principally cardiovascular disease, type 2 diabetes, and obesity—remain leading global drivers of premature mortality, disability, and healthcare expenditure, despite substantial advances in their biological understanding, risk assessment, and treatment. This narrative review critically examines why increasingly effective preventive interventions have failed to yield a commensurate reduction in population-level disease burden, identifying the biological, clinical, organizational, economic, and social factors that sustain this evidence-to-practice gap. It integrates evidence across three complementary dimensions: (1) The biological continuum, linking metabolic dysfunction, chronic low-grade inflammation, and cumulative exposure to apolipoprotein B (ApoB)-containing lipoproteins with atherosclerotic cardiovascular disease; (2) The clinical continuum, extending from early risk recognition and targeted diagnostic evaluation to individualized intervention and longitudinal reassessment; and (3) The organizational continuum, required to deliver coordinated, multidisciplinary prevention across health systems. Particular attention is given to total and lifetime cardiovascular risk, the clinical application and limitations of SCORE2 and SCORE2-OP, and the diagnostic challenges of identifying younger or apparently healthy individuals whose low short-term risk masks significant cumulative lifetime exposure. The review critically evaluates the causal role and clinical interpretation of ApoB-containing lipoproteins, including lipoprotein(a) [Lp(a)], and the complementary role of emerging biomarkers and subclinical atherosclerosis imaging, while carefully distinguishing enhanced risk prediction from demonstrated improvements in hard clinical outcomes. An educational and translational clinical vignette illustrates how the longitudinal interpretation of routine clinical and laboratory data supports proportionate assessment of persistent dyslipidemia, treatment response, and plausible primary and secondary contributors to cardiometabolic risk well before symptom onset. Rather than attributing borderline abnormalities to a single occult disorder, the vignette demonstrates how standardized confirmation, verification of medication exposure, etiological assessment, targeted referral, and structured reassessment can translate routine data into actionable preventive pathways. Ultimately, this analysis indicates that cardiometabolic prevention is constrained less by a lack of scientific evidence or effective pharmacotherapies than by delayed risk recognition, therapeutic inertia, poor treatment adherence, fragmented care pathways, limited multidisciplinary coordination, structural health inequalities, and the incomplete integration of clinical, laboratory, behavioral, environmental, and patient-reported data. Improving outcomes therefore demands a systemic shift from episodic, disease-specific management toward early, lifelong, proportionate, coordinated, equitable, and patient-centered cardiometabolic prevention—underpinned by implementation science, precision prevention, digital health solutions, health-economic evaluations, patient-reported outcome measures (PROMs), and patient-reported experience measures (PREMs).

## 1 Introduction

### 1.1 Cardiometabolic diseases as a system-wide public health challenge

Cardiometabolic diseases represent one of the major challenges facing contemporary healthcare systems, combining high prevalence, lifelong progression, escalating expenditures, and substantial preventable mortality (1–3). No longer confined to older populations or high-income nations, these conditions increasingly affect younger and working-age individuals, generating far-reaching clinical, social, and economic consequences (2, 3). Crucially, obesity, type 2 diabetes, dyslipidemia, hypertension, metabolic dysfunction-associated steatotic liver disease (MASLD), and cardiovascular disease are not isolated clinical entities; rather, they represent interconnected manifestations along a broader cardiometabolic spectrum.

This interdependence is driven by shared biological mechanisms. Chronic low-grade inflammation, impaired glucose and lipid homeostasis, insulin resistance, adipose tissue dysfunction, and endothelial injury interact over time, driving initially subtle and reversible metabolic disturbances toward progressive vascular damage and end-organ complications (4, 5). Obesity—particularly visceral adiposity—occupies a central position within this continuum. Its development reflects complex interactions among genetic susceptibility, aging, dietary patterns, physical inactivity, environmental factors, and altered adipokine signaling. These processes promote ectopic fat deposition, systemic insulin resistance, chronic inflammation, MASLD, atherogenic dyslipidemia, hypertension, and type 2 diabetes, significantly amplifying overall cardiovascular risk (5–7).

However, the existence of well-characterized biological mechanisms and effective preventive interventions has not translated into proportional improvements in population health. A substantial proportion of cardiometabolic disease remains attributable to modifiable or partially modifiable risk factors, including poor dietary habits, sedentary lifestyle, tobacco exposure, inadequate sleep, psychosocial stress, and broader social and commercial determinants of health. Despite increasingly sophisticated risk-prediction models, detailed clinical guidelines, advanced diagnostic and imaging tools, and highly effective therapies, preventive measures remain inconsistently implemented and unequally accessible (8–10).

This disconnect highlights a critical issue in modern medicine: the evidence-to-practice gap between established preventive strategies and their consistent execution in routine clinical care and public health. Consequently, the primary challenge is no longer merely generating additional biological knowledge or developing novel therapies, but ensuring that existing evidence is translated into timely, coordinated, equitable, and sustainable preventive action. Implementation science offers a valuable framework to address this gap by evaluating how organizational capacity, preventive infrastructure, therapeutic inertia, professional education, treatment adherence, reimbursement structures, and continuity of care affect the real-world impact of evidence-based recommendations. Recent European data reveal substantial heterogeneity in access to structured risk assessments, adoption of guideline-directed lipid-lowering therapies, multidisciplinary follow-up, and implementation of core European Society of Cardiology (ESC) recommendations across healthcare settings (9, 11, 12).

Within this framework, prevention must be conceptualized as a continuous, longitudinal process rather than a series of isolated consultations, tests, prescriptions, or disease-specific interventions. Its success depends on the ability of healthcare systems to detect risk early, interpret clinical data within context, initiate appropriate interventions, reassess risk over time, and sustain patient engagement throughout the care trajectory. Comprehensive care pathways linking primary care, laboratory medicine, subspecialty services, nursing, pharmacy, clinical nutrition, behavioral health, rehabilitation, and public health strengthen risk detection, care continuity, adherence, and long-term therapeutic efficacy (9, 13). Furthermore, this integrated approach enables the systematic collection of patient-reported outcome measures (PROMs) and patient-reported experience measures (PREMs) to identify and mitigate barriers related to communication, treatment burden, accessibility, and care coordination (10, 14).

Simultaneously, an integrated preventive model requires a critical evaluation of emerging tools proposed to refine cardiovascular risk assessment. Conventional risk algorithms often underestimate lifetime risk, particularly in younger populations, while novel biomarkers, genomic profiling, advanced imaging, and digital health tools remain incompletely integrated into clinical workflows. Enhanced predictive accuracy is clinically meaningful only if it alters management, improves patient understanding or adherence, and drives superior outcomes. Key questions surrounding incremental clinical utility, cost-effectiveness, population representativeness, equitable access, and the risk that digital or genomic innovations might exacerbate existing health disparities remain central to modern prevention (10, 15).

Accordingly, cardiometabolic prevention must be evaluated not only by the strength of its biological rationale or efficacy, but also by its feasibility, acceptability, long-term adherence, patient-reported outcomes, resource utilization, cost-effectiveness, scalability, continuity, and capacity to reduce health inequalities (12, 16). This perspective forms the conceptual foundation for this review, wherein biological, clinical, organizational, economic, technological, and public health determinants are examined as interconnected components of a unified preventive continuum.

### 1.2 The scale of the problem we can no longer ignore

Global epidemiological evidence highlights the scale of this challenge. Although age-standardized cardiovascular mortality has declined in several regions, cardiovascular diseases remain the leading cause of death worldwide. Meanwhile, the absolute number of individuals living with cardiovascular disease, experiencing acute events, or dying from cardiovascular causes continues to rise (17, 18). This growing burden unfolds alongside parallel surges in obesity and type 2 diabetes, creating an expanding population exposed to multiple interacting cardiometabolic risk factors across the lifespan (2, 17, 19).

The apparent paradox of declining age-standardized rates alongside an increasing absolute disease burden reflects major demographic and epidemiological transitions. Population growth and aging increase the number of individuals reaching ages where cardiovascular events are most frequent, while prolonged exposure to obesity, dyslipidemia, hypertension, hyperglycemia, and other modifiable risk factors steadily escalates cumulative biological risk (17, 18). Consequently, the public health impact of cardiometabolic disease cannot be adequately captured by short-term event rates alone; it must be evaluated in terms of millions living for decades with accumulating metabolic and vascular injury.

This burden is particularly acute in low- and middle-income settings, where rapid urbanization, changing food systems, physical inactivity, environmental exposures, and limited access to preventive healthcare drive adverse population-wide metabolic shifts (20, 21). These trends coexist with substantial disparities in healthcare infrastructure, workforce capacity, financing, health literacy, and service availability. Therefore, preventive strategies cannot simply be transferred across healthcare systems as uniform interventions. Their real-world effectiveness depends on tailored adaptation to local socioeconomic conditions, cultural contexts, service delivery models, financing structures, and healthcare accessibility (12, 22).

Type 2 diabetes exemplifies the trajectory of this growing global burden, with global prevalence projected to rise sharply over coming decades (2, 19). Obesity serves as a primary upstream driver of this expansion and is strongly linked to hypertension, dyslipidemia, insulin resistance, type 2 diabetes, MASLD, and accelerated atherogenesis (5–7). These sequelae extend beyond excess body weight alone; they reflect persistent metabolic, inflammatory, endocrine, and vascular disturbances interacting across the life course (4, 5).

Visceral adipose tissue, in particular, is now recognized as an active immunometabolic and endocrine organ rather than a passive energy reservoir. Through adipokines, inflammatory cytokines, and bioactive mediators, dysfunctional adipose tissue fosters chronic low-grade inflammation, insulin resistance, mitochondrial dysfunction, endothelial injury, and systemic metabolic dysregulation (5, 23). These mechanisms provide a unified biological substrate connecting obesity and type 2 diabetes with MASLD, atherosclerosis, and overt cardiovascular disease (5–7, 23).

A critical common denominator across these conditions is the prolonged convergence of hyperglycemia, elevated blood pressure, insulin resistance, and increased circulating concentrations of atherogenic apolipoprotein B (ApoB)-containing lipoproteins. These abnormalities act synergistically rather than in isolation. Over time, they promote endothelial activation, subendothelial retention and modification of ApoB-containing particles, vascular inflammation, and progressive atherosclerotic plaque evolution—directly bridging early metabolic dysfunction with clinical cardiovascular events (4, 5, 24).

Encouragingly, substantial components of this burden are modifiable. Interventional data demonstrate that structured lifestyle modification, contemporary anti-obesity pharmacotherapy, and bariatric surgery yield meaningful improvements in body weight, glycemic control, systemic inflammation, lipid profiles, and overall cardiovascular risk (6, 23, 25, 26). Randomized controlled trials further show that combining contemporary anti-obesity pharmacotherapy—specifically glucagon-like peptide-1 (GLP-1) receptor agonists—with structured lifestyle interventions results in greater weight loss and broader improvements in cardiometabolic risk factors than lifestyle measures alone (26).

However, the population-level impact of these therapies depends on factors far beyond biological efficacy. Equitable access, affordability, tolerability, long-term adherence, behavioral support, and structured follow-up dictate whether efficacy observed in clinical trials translates into sustained effectiveness in routine practice (9, 12, 26). Lifestyle modification thus remains the cornerstone of cardiometabolic prevention, while pharmacological and surgical strategies serve as essential complementary tools within a comprehensive long-term management plan (10, 26). Furthermore, individual capacity to modify cardiometabolic risk is heavily conditioned by socioeconomic status, commercial food environments, occupational stressors, health literacy, healthcare access, and ongoing clinical support (9, 10, 12, 22). Sustained preventive benefit therefore requires embedding evidence-based interventions within longitudinal care pathways and supportive environments (9, 13, 14).

Despite the magnitude of this burden and the expanding therapeutic armamentarium, the scientific literature remains largely siloed around individual diseases, single risk factors, isolated biomarkers, or specific therapeutic modalities. Extensive evidence addresses obesity, type 2 diabetes, atherogenic dyslipidemia, risk prediction models (27, 28), ApoB, lipoprotein(a), vascular imaging, lifestyle interventions, and precision medicine (15, 24, 27, 28). What remains underdeveloped is their integration at the critical interfaces where biological mechanisms, laboratory diagnostics, patient behavior, clinical decision-making, and health systems organization intersect throughout the patient journey (8–10). This fragmentation hampers the translation of robust evidence into coherent, lifelong preventive strategies.

Key operational uncertainties also persist regarding the real-world implementation of modern advances. These include determining how lifetime risk assessment should complement established short-term risk estimation in routine clinical practice; appropriately incorporating family history, polygenic risk scores, coronary artery calcium scoring, ApoB, lipoprotein(a), and novel inflammatory biomarkers into clinical practice; and evaluating the clinical utility, cost-effectiveness, scalability, and equitable deployment of precision and digital health technologies (11, 12, 15, 27–32). Statistical refinement alone will not reduce disease burden if individuals at elevated risk remain unidentified, undertreated, lost to follow-up, or disengaged from shared decision-making. The fundamental question is not merely whether a novel biomarker, imaging tool, or predictive model enhances risk classification, but whether its implementation drives clinically meaningful, equitable, cost-effective, and sustainable improvements in patient-centered outcomes (15, 27–32).

To address these gaps, this review conceptualizes cardiometabolic prevention as an integrated, lifelong, and system-wide framework rather than a fragmented set of disease-specific guidelines. We synthesize three interconnected dimensions:

1. The biological continuum: Linking early metabolic dysfunction, chronic inflammation, and cumulative exposure to ApoB-containing lipoproteins with atherosclerotic cardiovascular disease;
2. The clinical continuum: Extending from early risk detection, laboratory interpretation, multimodal risk stratification, inherited risk assessment, and individualized treatment escalation to longitudinal monitoring (15);
3. The organizational continuum: Delivering coordinated, patient-centered prevention through integrated multidisciplinary care pathways (9, 14).

Moving beyond disease-centric paradigms, we propose an integrative conceptual framework bridging biological evidence, implementation science, and health services delivery. Within this framework, we critically evaluate the limitations of short-term risk prediction, the pragmatic utility of emerging biomarkers, therapeutic inertia, non-adherence, care fragmentation, digital exclusion, health disparities, and the long-term sustainability of preventive interventions. Ultimately, this work aims to bridge the evidence-to-practice gap and support a transition from episodic disease management toward coordinated, lifelong cardiometabolic prevention toward coordinated, lifelong cardiometabolic prevention based on shared responsibility among patients, healthcare professionals, and health systems (8–10, 14, 15). The subsequent sections explore this continuum—from underlying biological mechanisms and advanced risk assessment to implementation science, integrated care models, and future directions in precision prevention (14, 15).

### 1.3 Literature search approach

To ensure methodological transparency, this narrative review was informed by a structured literature search conducted primarily across PubMed/MEDLINE, Scopus, and Web of Science. The search strategy focused on literature relevant to the biological, clinical, and health-system dimensions of cardiometabolic prevention. Search terms and their combinations included: *cardiometabolic disease*, *cardiovascular prevention*, *obesity*, *type 2 diabetes*, *atherosclerosis*, *dyslipidemia*, *apolipoprotein B* (*ApoB*), *lipoprotein(a)* (*Lp(a)*), *SCORE*, *SCORE2*, *SCORE2-OP*, *lifetime cardiovascular risk*, *coronary artery calcium*, *cardiovascular imaging*, *metabolic dysfunction-associated steatotic liver disease* (*MASLD*), *thyroid dysfunction*, *implementation science*, *integrated care*, *multidisciplinary care*, *treatment adherence*, *patient-reported outcome measures*, *patient-reported experience measures*, *digital health*, *precision prevention*, *health inequalities*, and *health economics*. Boolean operators and combinations of related terms were applied to identify literature addressing the intersections among these domains.

The primary search targeted peer-reviewed publications available through August 2026, with particular emphasis on recent international and European clinical guidelines, scientific statements, consensus documents, large epidemiological studies, randomized controlled trials, systematic reviews, and contemporary implementation research. Earlier landmark publications were retained to document the historical evolution of cardiovascular risk assessment or to support well-established pathophysiological concepts that remain fundamental to current preventive practice. In addition, reference lists of major guidelines, consensus statements, and highly relevant reviews were hand-searched to identify pertinent primary studies and authoritative sources not captured by the initial database queries.

Study selection was guided by relevance to the conceptual framework of this review, methodological quality, clinical applicability, and contribution to the biological, diagnostic, therapeutic, organizational, economic, or implementation dimensions of cardiometabolic prevention. Particular priority was given to evidence addressing the translation of established preventive knowledge into routine clinical practice, including barriers to risk identification, treatment initiation and intensification, long-term adherence, multidisciplinary coordination, equitable access, digital implementation, and continuity of care.

Because this work was designed as a narrative rather than a systematic review, formal PRISMA-based screening, predefined quantitative inclusion and exclusion algorithms, risk-of-bias assessments, and meta-analytic syntheses were not performed. The search strategy was intended to provide a transparent, structured foundation for a critical integrative synthesis rather than an exhaustive systematic identification of all publications across each individual domain.

## 2 Assessment of total cardiovascular risk: the foundation of modern prevention

### 2.1 The concept of total cardiovascular risk

Total cardiovascular risk constitutes the cornerstone of contemporary cardiovascular prevention (33, 34). It recognizes that cardiovascular disease results from the cumulative interaction of biological, behavioral, environmental, and health-system determinants rather than from isolated risk factors alone (33). Consequently, the estimated 10-year probability of a fatal or non-fatal cardiovascular event should be interpreted as only one component of a broader lifelong cardiometabolic risk trajectory rather than a complete representation of an individual’s future cardiovascular risk (27, 33).

Importantly, this concept extends beyond the variables incorporated into conventional risk-prediction models. Cardiovascular risk reflects the cumulative consequences of lifelong exposure to interacting biological, behavioral, environmental, socioeconomic, and healthcare-system factors. Modern prevention therefore demands a transition from disease-specific management toward an integrated cardiometabolic paradigm that combines rigorous biological risk assessment with implementation strategies capable of translating evidence into routine clinical workflows (8, 10, 24).

This holistic risk perspective is strongly supported by the epidemiology of non-communicable lifestyle diseases (4, 35). Obesity, type 2 diabetes, hypertension, and atherogenic dyslipidemia rarely occur in isolation; more frequently, they present as an interconnected cluster of cardiometabolic risk factors (1, 4). Although hypertension is not a primary metabolic disorder per se, it is intimately linked to visceral obesity, insulin resistance, and dyslipidemia, constituting an integral component of the cardiometabolic disease spectrum (4, 5). The coexistence of these conditions synergistically accelerates atherogenesis and amplifies the risk of clinical complications; evaluating a single risk factor in isolation therefore fails to capture the true disease burden (4, 15). This biological clustering explains why disease-specific approaches may underestimate cumulative cardiometabolic harm and why integrated prevention has become a central objective of contemporary clinical guidelines (10, 24).

According to European Society of Cardiology (ESC) and European Atherosclerosis Society (EAS) guidelines, the intensity of preventive interventions should be proportional to total cardiovascular risk: the higher the overall risk, the earlier, more intensive, and more comprehensive the preventive intervention should be (33, 34, 36). In practice, this approach moves preventive care away from rigid thresholds based on single laboratory values or isolated blood pressure measurements toward strategies aimed at reducing overall cardiovascular risk.

In individuals with established cardiovascular disease or specific high-risk conditions—including selected patients with diabetes mellitus, chronic kidney disease, or markedly elevated individual risk factors—formal risk estimation using SCORE2 may be unnecessary because their clinical profile automatically places them in a high or very high cardiovascular risk category (33, 34). Conversely, in apparently healthy individuals without established high-risk conditions, validated risk-estimation algorithms—specifically SCORE2 and SCORE2-OP—are recommended to support cardiovascular risk stratification (27, 28, 36). These tools facilitate the identification of individuals who, despite lacking overt cardiovascular symptoms, may derive substantial benefit from appropriately targeted preventive interventions (15, 33, 36).

### 2.2 Risk assessment systems and their clinical significance

The formal assessment of total cardiovascular risk serves as a vital link between population-level epidemiology and everyday clinical practice (33, 36). Its primary purpose is not merely to assign a patient to a static risk category, but to facilitate rational, risk-proportionate, and effective preventive care (33, 34, 36). Risk assessment algorithms should be understood as clinical decision-support tools that inform, rather than dictate, therapeutic choices (36). In this capacity, they represent the practical translation of epidemiological evidence into individualized patient care (33, 36). Accordingly, cardiovascular risk models should complement, rather than replace, clinical reasoning, contextual risk interpretation, and shared decision-making (9, 33, 36).

Their principal value lies in facilitating structured risk communication between healthcare professionals and patients, supporting shared decision-making, and identifying individuals who are most likely to benefit from timely preventive interventions, rather than providing rigid, automated treatment directives (10, 15).

#### 2.2.1 The SCORE system as the basis for risk stratification in Europe

The original Systematic Coronary Risk Estimation (SCORE) system was designed to estimate the 10-year risk of fatal cardiovascular events, as reflected in earlier European guidelines (37). Derived from large European cohort studies, SCORE served for many years as the principal framework for cardiovascular risk stratification in Europe. Changes in cardiovascular epidemiology, improvements in prevention and treatment, and the need to account for clinically relevant non-fatal cardiovascular events subsequently led to the development of the SCORE2 algorithm (27).

The transition from SCORE to SCORE2 represents an important conceptual and methodological advance in preventive cardiology, extending risk prediction beyond cardiovascular mortality to encompass the 10-year probability of a first fatal or non-fatal cardiovascular event (27, 37). Improved survival following cardiovascular events has increased the clinical importance of non-fatal outcomes, reinforcing the need for prediction models that capture both fatal and non-fatal events (18, 27). Nevertheless, SCORE2 should be interpreted as one component of a broader clinical evaluation rather than a comprehensive or definitive measure of an individual’s cardiometabolic risk (15, 27).

SCORE2 is designed primarily for cardiovascular risk estimation in apparently healthy individuals aged 40–69 years, whereas SCORE2-OP extends the framework to older adults aged 70 years and above (27, 28). Both models estimate the 10-year risk of a first fatal or non-fatal cardiovascular event using routinely available clinical parameters, including age, sex, smoking status, systolic blood pressure, and non-HDL cholesterol (27, 28).

A major strength of the SCORE2 framework is its recalibration across European risk regions according to underlying patterns of cardiovascular incidence and mortality. This regional approach reduces systematic overestimation or underestimation of risk resulting from substantial differences in baseline cardiovascular disease burden across European populations (27, 33).

Importantly, neither SCORE2 nor SCORE2-OP provides a permanent risk assignment. Rather, the estimated risk represents a time-specific assessment within a dynamic preventive strategy that requires periodic reassessment as age, risk-factor exposure, comorbidities, treatment response, and other relevant clinical circumstances evolve across the life course.

#### 2.2.2 Limitations of point-based models and the need for clinical interpretation

Despite their broad clinical utility, SCORE2 and SCORE2-OP have important limitations. SCORE2 is intended primarily for apparently healthy individuals aged 40–69 years, whereas SCORE2-OP addresses older adults; nevertheless, both models necessarily incorporate a restricted set of variables and therefore cannot capture all clinically relevant factors that may modify individual cardiovascular susceptibility (27, 28, 33).

Recent studies have also highlighted practical implementation challenges associated with SCORE2. Simplified paper-based risk charts approximate continuous underlying risk equations by grouping continuous clinical variables into predefined categories. Consequently, the same individual may be assigned to a different cardiovascular risk category depending on whether risk is estimated using a printed chart or a validated digital calculator implementing the continuous mathematical model (38, 39). Such discrepancies may directly influence treatment eligibility, particularly in individuals whose estimated risk lies close to age-specific therapeutic decision thresholds. Whenever feasible, validated digital calculators implementing the continuous SCORE2 equations should therefore be prioritized, particularly in borderline cases. These observations illustrate that even highly validated risk models are influenced by their mode of clinical implementation and that implementation fidelity can directly affect preventive decision-making (39).

Beyond these operational considerations, cardiovascular risk estimates may be affected by changes in underlying population epidemiology. Risk models can become progressively less well calibrated when cardiovascular incidence, mortality, treatment patterns, or the prevalence of metabolic risk factors change substantially over time (27, 33, 36). For this reason, final cardiovascular risk assessment should incorporate relevant risk modifiers and the broader clinical context rather than relying exclusively on the numerical output of a prediction model (33, 36).

Current European guidelines and contemporary evidence emphasize the importance of considering additional factors that may modify estimated cardiovascular risk, including adiposity, physical inactivity, psychosocial factors, family history of premature cardiovascular disease, chronic inflammatory conditions, chronic kidney disease, and other clinically relevant comorbidities (33). Where appropriate, additional metabolic or organ-specific conditions may further inform individual risk interpretation. The contribution of such modifiers should be considered in the context of the patient’s baseline estimated risk and only when their inclusion is likely to influence clinical management.

This necessity underscores a fundamental principle of contemporary preventive cardiology: final risk stratification must integrate clinical judgment and individual patient context rather than rely uncritically on algorithmic outputs (33, 36).

Moreover, because SCORE2 estimates cardiovascular risk over a 10-year horizon, it may underestimate the long-term consequences of sustained adverse risk-factor exposure in younger adults, particularly those with severe dyslipidemia, obesity, type 2 diabetes, strong familial predisposition, elevated lipoprotein(a) [Lp(a)], high apolipoprotein B (ApoB) concentrations, or prolonged exposure to adverse metabolic environments (15, 27, 32, 33, 40). These limitations align with the emerging concept of lifetime cardiovascular risk, which reinforces the need for personalized interpretation extending beyond short-term mathematical estimates (15, 24). This consideration is particularly important because cumulative subclinical vascular injury may begin decades before short-term 10-year risk reaches conventional intervention thresholds.

#### 2.2.3 Risk reclassification and the role of subclinical atherosclerosis markers

Determining optimal management can be particularly challenging in individuals whose estimated cardiovascular risk lies close to a clinical decision threshold. SCORE2-based risk categories are age-dependent and are used to support classification into low-to-moderate, high, or very high cardiovascular risk groups (27, 28, 33).

In individuals at low-to-moderate or borderline estimated risk, conventional risk assessment may leave uncertainty regarding the expected benefit of initiating or intensifying preventive pharmacotherapy. In such circumstances, selected additional investigations may improve risk characterization when their results are likely to alter clinical management (33, 36).

Among these approaches, direct assessment of subclinical atherosclerosis can provide clinically relevant information in appropriately selected individuals. Available modalities include coronary artery calcium (CAC) scoring using non-contrast computed tomography and vascular ultrasound for the detection of carotid or femoral atherosclerotic plaques (33). The ankle–brachial index may also provide additional evidence of peripheral artery disease in appropriate clinical settings (33). A CAC score >100 Agatston units provides substantial risk-reclassification value and identifies individuals with a markedly increased risk of future cardiovascular events beyond that predicted by traditional risk factors (30, 41).

However, although CAC scoring, vascular ultrasound, ApoB, Lp(a), novel inflammatory biomarkers, and polygenic risk scores demonstrate potential for refining cardiovascular risk assessment, important questions remain regarding their optimal clinical sequencing, cost-effectiveness, equitable access, and ultimate effect on hard clinical outcomes. Supplementary testing should therefore be considered primarily when its result has a reasonable probability of changing clinical management rather than merely improving statistical risk classification (10, 15). The objective of advanced risk assessment is not to increase diagnostic complexity, but to identify individuals for whom more precise risk characterization can meaningfully inform preventive decisions (10, 15).

Conversely, downward risk reclassification can also be clinically informative. The absence of detectable coronary artery calcification (CAC = 0) is associated with a low short-to-medium-term risk of cardiovascular events in appropriately selected individuals and may support a more conservative approach to the initiation of lipid-lowering pharmacotherapy when no major competing risk modifiers are present (30, 41). Importantly, CAC = 0 should not be interpreted as evidence of the complete absence of subclinical atherosclerosis or as a guarantee of negligible lifetime cardiovascular risk. Its clinical significance must therefore be interpreted alongside age, cumulative risk-factor exposure, family history, and other relevant risk modifiers.

#### 2.2.4 The significance of risk categories for prevention strategies

Although total cardiovascular risk exists along a continuous spectrum, categorical risk stratification remains clinically useful for guiding the intensity of preventive interventions (27, 28, 33, 34). Individuals classified as being at high or very high cardiovascular risk generally require early and intensive preventive strategies that combine lifestyle intervention with evidence-based pharmacotherapy directed toward appropriate risk-factor targets (33, 34).

At the same time, individuals classified as being at low-to-moderate short-term risk represent an important population for primary prevention. In this group, particularly among younger adults, apparently reassuring short-term risk estimates may coexist with prolonged exposure to adverse metabolic factors and a potentially substantial lifetime cardiovascular burden. This population therefore offers an important opportunity to prevent or delay the progression of subclinical cardiometabolic disease through timely lifestyle modification, management of modifiable metabolic risk factors, and, where clinically indicated, pharmacological interventions before irreversible vascular damage develops (19, 33, 34, 42).

Consequently, modern cardiovascular prevention should not be conceptualized as a static, one-off process of assigning an individual to a risk category. Instead, cardiovascular risk assessment should form part of an ongoing, adaptive preventive pathway incorporating repeated biological and clinical reassessments, longitudinal monitoring of risk-factor trajectories, sustained patient engagement, shared decision-making, and coordinated care across the lifespan (8, 10). Within this framework, risk categories provide a practical basis for clinical action, but their value ultimately depends on whether they are translated into timely, proportionate, and sustained preventive interventions.

## 3 From data to evidence: the role of lipids in the pathophysiology of atherosclerosis

### 3.1 LDL and ApoB-containing lipoproteins as causal factors in atherosclerosis

Contemporary epidemiological, clinical, and genetic evidence consistently supports a causal role of apolipoprotein B (ApoB)-containing lipoproteins in atherosclerotic cardiovascular disease (ASCVD) (40). Converging evidence from human genetics, observational epidemiology, and randomized controlled trials demonstrates that these atherogenic particles initiate and accelerate the atherosclerotic process (40). Atherosclerosis represents the principal pathological substrate underlying myocardial infarction and ischemic stroke, with its development and progression determined by both the magnitude and cumulative duration of arterial exposure to circulating atherogenic lipoproteins (24, 40).

The initial identification of cholesterol within human atheromatous plaques led to the classic “cholesterol hypothesis.” Contemporary lipidology, however, establishes that the primary causal determinant is not cholesterol mass in isolation, but rather the circulating burden of ApoB-containing lipoprotein particles capable of penetrating and becoming retained within the arterial intima (40, 43). Population-based studies consistently demonstrate that elevated low-density lipoprotein cholesterol (LDL-C) and apolipoprotein B100 (ApoB)—the essential structural protein present on each atherogenic particle—are strongly associated with an increased incidence of atherothrombotic cardiovascular events (31, 40).

This distinction has important clinical implications. LDL-C quantifies the cholesterol mass carried within LDL particles, whereas ApoB concentration provides a clinically robust estimate of the number of circulating atherogenic particles. Although these two measures are frequently concordant, clinically relevant discordance is common in individuals with hypertriglyceridemia, visceral obesity, insulin resistance, metabolic syndrome, or type 2 diabetes. In these cardiometabolic phenotypes, a deceptively modest LDL-C concentration may coexist with a high burden of ApoB-containing particles; consequently, reliance on LDL-C alone may underestimate residual atherogenic risk (31, 40).

ApoB-containing lipoproteins of sufficiently small particle size—including LDL, IDL, triglyceride-rich remnant particles, Lp(a), and smaller VLDL particles—can cross the vascular endothelial barrier and become retained within the subendothelial intima (32, 43). Subendothelial retention and entrapment of these particles constitute a key initiating event in atherogenesis. This process can be amplified by endothelial dysfunction, which increases vascular permeability and facilitates interactions between ApoB-containing lipoproteins and extracellular matrix proteoglycans (24, 29, 43).

Once retained within the arterial wall, lipoproteins undergo structural and biochemical modifications, most notably enzymatic and oxidative modification (24, 29, 43). The susceptibility of retained particles to subendothelial modification may be further influenced by systemic metabolic and endocrine disturbances. For example, overt or subclinical thyroid dysfunction can alter hepatic lipoprotein receptor regulation, impair LDL clearance, adversely affect endothelial function, and contribute to increased long-term cardiovascular risk—mechanisms explored further in subsequent sections of this review (44–47).

Native LDL particles are not efficiently internalized by macrophages; however, their subendothelial modification promotes recognition by scavenger receptors and the formation of highly atherogenic modified LDL species, including oxidized LDL (oxLDL) (24, 29, 43). Concurrently, endothelial activation increases the expression of adhesion molecules, facilitating monocyte recruitment into the subendothelial space, where these cells differentiate into tissue macrophages. Unregulated scavenger receptor-mediated uptake of modified ApoB-containing lipoproteins subsequently promotes intracellular lipid accumulation and foam-cell formation, a pathological hallmark of early atherosclerotic fatty streaks (24, 29, 43, 48).

Activated foam cells and tissue macrophages sustain a local pro-inflammatory cascade that promotes further lipoprotein retention, monocyte recruitment, and plaque progression. In parallel, inflammatory mediators stimulate the migration and proliferation of vascular smooth muscle cells (VSMCs). These cells synthesize extracellular matrix components, including interstitial collagen, contributing to the formation of a fibrous cap over the lipid-rich necrotic core. Plaque stability therefore reflects a dynamic balance among persistent inflammatory activity, extracellular matrix synthesis and degradation, and the reparative capacity of vascular smooth muscle cells (24, 29, 43, 48).

Persistent and uncontrolled local inflammation can shift plaque biology toward an unstable phenotype characterized by an expanding necrotic core, progressive collagen degradation, thinning of the fibrous cap, thin-cap fibroatheroma (TCFA) formation, and intraplaque hemorrhage. Plaque rupture or superficial erosion exposes thrombogenic components to circulating blood, promoting rapid platelet activation and coagulation, potentially culminating in acute luminal thrombosis and clinically manifest ischemic events (24, 29, 48).

Crucially, atherosclerosis is not the consequence of a single acute physiological insult, but rather develops through cumulative exposure of the arterial wall to circulating ApoB-containing lipoproteins over time (40). The higher the circulating concentration of these particles and the longer the duration of vascular exposure, the greater the probability of their arterial retention and the more rapidly total atherosclerotic plaque burden accumulates.

Atherosclerosis is therefore best conceptualized as a cumulative exposure process in which both atherogenic particle burden and duration of exposure contribute to lifetime arterial disease burden. Within this framework, ApoB provides a biologically relevant estimate of atherogenic particle number, whereas LDL-C remains the principal and extensively validated therapeutic target, supported by randomized controlled trial evidence and incorporated into contemporary clinical guidelines (34, 40). These laboratory parameters should therefore be regarded as complementary rather than mutually exclusive clinical tools.

Although ApoB provides a biologically informative measure of atherogenic particle burden, its translation into routine clinical practice illustrates a broader challenge in preventive cardiology. The availability of a potentially more informative biomarker does not automatically improve patient outcomes unless the test is appropriately ordered, accurately interpreted, incorporated into clinical decision-making, and linked to timely and effective therapeutic action. Distinguishing analytical and prognostic performance from demonstrated real-world clinical utility is therefore an important principle of implementation science in cardiovascular medicine (8–10).

Direct measures or estimates of atherogenic particle number, including ApoB concentration and LDL particle number (LDL-P), may improve cardiovascular risk characterization compared with LDL-C alone, particularly in states of discordance (31, 40). Non-high-density lipoprotein cholesterol (non-HDL-C) also provides a practical and inexpensive complementary measure because it reflects the cholesterol content carried by the full spectrum of atherogenic ApoB-containing lipoproteins and can be readily calculated from a standard lipid profile (34, 43, 49–51). Epidemiological evidence further supports the association of elevated non-HDL-C with subsequent coronary heart disease and selected stroke outcomes (51).

Nevertheless, the biological and prognostic advantages of ApoB as a marker of atherogenic particle burden do not automatically resolve how it should be operationalized in everyday clinical practice. Important uncertainties remain regarding which patient subgroups derive sufficient incremental clinical benefit from routine ApoB testing, the therapeutic decision thresholds at which ApoB should trigger pharmacological intensification, assay standardization across laboratories, health-economic implications, and whether systematic ApoB-guided management produces additional reductions in hard clinical outcomes beyond established LDL-C and non-HDL-C treatment strategies. Accordingly, broader implementation of ApoB testing should be accompanied by prospective research evaluating clinical utility, cost-effectiveness, health equity, and real-world treatment adherence rather than relying solely on incremental improvements in statistical risk discrimination or reclassification (9, 10, 16, 51).

The substantial efficacy of pharmacological LDL-C lowering—most notably with statins, ezetimibe, and PCSK9-targeted therapies—in reducing major adverse cardiovascular events (MACE) provides compelling clinical evidence supporting the causal role of ApoB-containing lipoproteins in atherosclerosis (34, 40). Accumulating evidence further indicates that other ApoB-containing particles, including triglyceride-rich remnant lipoproteins and Lp(a), contribute to residual cardiovascular risk in treated individuals (32, 40, 43).

However, biological causality does not imply that identical therapeutic strategies should be applied to all ApoB-containing lipoproteins. Whereas LDL-C lowering is supported by decades of randomized cardiovascular outcome trials, therapeutic strategies specifically targeting remnant lipoproteins, ApoB discordance, or elevated Lp(a) are less uniformly established. Mechanistic plausibility and epidemiological association should therefore not be conflated with demonstrated clinical efficacy until dedicated intervention trials establish meaningful reductions in hard clinical outcomes (32, 34).

In summary, cumulative exposure to ApoB-containing lipoproteins provides a strong biological rationale for early, intensive, and sustained lipid-lowering strategies when clinically indicated (34, 40). Yet translating these pathophysiological insights into population-level reductions in ASCVD burden requires considerably more than selecting an optimal biomarker or pharmacological agent. It requires timely risk recognition, appropriate treatment initiation and intensification, long-term adherence, continuous monitoring, equitable access to effective therapies, and coordinated care across primary and specialist services. Successful lipid management depends not only on selecting appropriate biomarkers and treatments, but also on maintaining care continuity and sustained patient engagement throughout the preventive pathway (9, 12, 14, 16, 50). Bridging this persistent evidence-to-practice gap remains as fundamental to improving cardiovascular health as understanding the underlying mechanisms of atherogenesis itself (8–10).

### 3.2 Lipoprotein(a): an underestimated and frequently overlooked risk factor

A distinct and historically underrecognized member of the ApoB-containing lipoprotein family is lipoprotein(a) [Lp(a)], an atherogenic particle composed of an LDL-like core containing ApoB100 covalently bound to the specialized glycoprotein apolipoprotein(a). Like LDL, Lp(a) can cross the endothelial barrier and become retained within the arterial wall; however, its pathogenic properties extend beyond cholesterol delivery alone (32, 43).

The pathogenicity of Lp(a) reflects both its ApoB-containing lipoprotein core and the distinctive structural and biological properties of apolipoprotein(a), which confer pro-atherogenic, pro-inflammatory, and potentially pro-thrombotic effects. The structural homology between apolipoprotein(a) and plasminogen has led to the hypothesis that Lp(a) may interfere with fibrinolytic pathways, while its substantial content of oxidized phospholipids (OxPL) contributes to vascular inflammation, endothelial dysfunction, and the progression of atherosclerotic lesions (24, 29, 32, 43).

Circulating Lp(a) concentrations are predominantly genetically determined, with most interindividual variability attributable to variation at the *LPA* gene locus, and levels generally remain relatively stable throughout adult life (32). Consequently, markedly elevated Lp(a) can confer substantial cumulative lifetime ASCVD risk long before overt clinical symptoms or detectable manifestations of advanced vascular disease emerge. Very high Lp(a) concentrations, particularly levels >180 mg/dL (>430 nmol/L), have been associated with a lifetime cardiovascular risk comparable to that observed in heterozygous familial hypercholesterolemia (FH) (15, 32–34).

However, the general principle that Lp(a) concentrations remain relatively stable throughout life is not absolute. Circulating levels may be influenced by acute systemic inflammation, advanced renal disease, pregnancy, menopause, and selected hepatic or endocrine disorders. Repeat testing may therefore be clinically appropriate when the initial measurement is obtained during an acute-phase response or appears inconsistent with the broader clinical context (32, 34).

Conceptually, Lp(a) should not be regarded merely as an additional component of the lipid profile, but rather as an important modifier of vascular risk capable of amplifying atherogenic, inflammatory, oxidative, and potentially thrombotic processes within the arterial wall (24, 29, 32).

Accordingly, current European guidelines recommend measuring Lp(a) at least once during adulthood to identify individuals with genetically determined elevations that may not be adequately captured by conventional cardiovascular risk algorithms (33, 34). Despite this recommendation, implementation in routine clinical practice remains inconsistent across health systems. In many settings, Lp(a) testing remains underutilized in primary care, creating missed opportunities for earlier risk recognition, family-based cascade testing, and more individualized preventive management (9, 12).

Although this recommendation represents an important advance in preventive cardiology, several barriers continue to impede its routine implementation. In addition to low testing rates, interpretation is complicated by differences in laboratory reporting using mass units (mg/dL) or molar concentrations (nmol/L). Because variation in apolipoprotein(a) isoform size prevents the use of a universally valid mathematical conversion factor between these units, standardized and clinically interpretable laboratory reporting is essential. Broader implementation therefore requires improved assay standardization, harmonized reporting practices, clinician education, and appropriate integration of Lp(a) measurements into electronic clinical decision-support systems (9, 12).

A further clinical challenge is that the identification of markedly elevated Lp(a) currently exceeds the availability of targeted therapies with definitively established cardiovascular outcome benefits in routine clinical practice. Novel RNA-targeted approaches—including antisense oligonucleotide and small interfering RNA strategies—are being evaluated in advanced cardiovascular outcome programs. Until targeted Lp(a)-lowering therapies demonstrate definitive outcome benefits and become incorporated into routine evidence-based practice, clinical management should focus on intensive control of concomitant modifiable cardiovascular risk factors, including LDL-C, blood pressure, glycemia, smoking status, body weight, and physical activity (33, 34). This requires careful risk communication: clinicians should explain that elevated Lp(a) represents a lifelong risk-enhancing factor rather than an untreatable disease and that its detection should prompt comprehensive global risk reduction rather than therapeutic fatalism.

The population-level health-economic value of universal compared with targeted Lp(a) screening also remains incompletely established. Future implementation studies should determine whether universal adult measurement improves treatment initiation, cascade family testing, long-term medication adherence, and ultimately the incidence of hard clinical outcomes, and whether these benefits justify the associated organizational and financial costs across diverse health systems (10, 15, 16).

Collectively, these observations reinforce a central premise of this review: advances in preventive cardiology should not be evaluated solely according to the discovery or analytical performance of novel biomarkers, but also according to their capacity to improve real-world patient care. Lp(a) provides a particularly instructive example of how biological insight, laboratory diagnostics, risk communication, family-based screening, multidisciplinary management, and healthcare delivery must operate within an integrated preventive continuum. Only through such integration can improved identification of genetically mediated cardiovascular risk be translated into earlier intervention, sustained patient engagement, greater health equity, and measurable reductions in population cardiovascular burden (8–10, 12).

## 4 Chapter 4: Prevention and treatment targets—from lifestyle intervention to combination therapy

### 4.1 Non-pharmacological prevention as the foundation

Understanding the biology of atherosclerosis leads to a fundamental conclusion: prevention is not an adjunct to treatment—it is its foundation. Because total atherosclerotic burden depends on cumulative arterial exposure to atherogenic lipoproteins and other cardiometabolic risk factors, interventions that reduce this exposure yield substantial benefits when initiated early and maintained over time (33, 34, 40). From a public health perspective, preventive strategies must begin before the onset of overt cardiovascular disease, while modifiable risk factors are still accumulating (4, 10, 33).

ESC prevention guidelines emphasize the maintenance of optimal cardiovascular health across the lifespan. Key priorities include avoiding tobacco use, engaging in regular physical activity, adopting healthy dietary patterns, maintaining an optimal body weight, and effectively managing blood pressure, glycemia, and atherogenic lipoproteins (33, 34). Rather than addressing these factors in isolation, contemporary prevention aims to reduce their combined contribution to total and lifetime cardiovascular risk (4, 33).

In individuals with overweight or obesity, sustained weight reduction of 5–10%, combined with 150–300 min of moderate-intensity physical activity per week and adherence to a healthy dietary pattern, can concurrently improve multiple cardiometabolic parameters (23, 26, 33). These interventions can reduce blood pressure, improve insulin sensitivity and glycemic control, decrease visceral adiposity, and favorably modify circulating atherogenic lipoproteins (4, 7, 23, 26).

However, lifestyle modification should not be viewed merely as a matter of individual choice or personal responsibility. Dietary habits, physical activity, smoking status, and treatment adherence are strongly influenced by socioeconomic status, health literacy, occupational demands, food environments, access to safe recreational spaces, cultural norms, and the structure of health systems (9, 11, 12, 21). Consequently, effective prevention requires both individualized clinical counseling and population-level policies that make healthy choices accessible, affordable, and sustainable (9, 11, 12).

Lifestyle interventions are most effective when delivered through coordinated care pathways involving primary care physicians, nurses, dietitians, psychologists, pharmacists, rehabilitation specialists, and public health professionals. Their success depends on care continuity, sustained behavioral reinforcement, and active patient participation in shared decision-making throughout the preventive continuum (9, 12, 14).

A major challenge is that the benefits of lifestyle modification are often gradual and less immediately visible than those of pharmacotherapy. This delayed visibility may diminish patient motivation and contribute to therapeutic inertia when clinicians or patients expect rapid improvements in laboratory parameters or symptoms. Nevertheless, even modest improvements sustained over decades can translate into meaningful reductions in lifetime cardiovascular risk (33, 40).

The effectiveness of lifestyle counseling depends substantially on how it is delivered. Brief, isolated advice provided during a single consultation rarely produces durable behavioral change. More effective strategies combine structured follow-up, shared decision-making, clear and realistic goal setting, dietary and behavioral coaching, multidisciplinary support involving nurses, dietitians, pharmacists, and rehabilitation specialists, and appropriately implemented digital tools such as automated reminders or remote monitoring (9, 11, 14).

Equally important is the systematic evaluation of patient-reported outcome measures (PROMs) and patient-reported experience measures (PREMs). Regular feedback regarding communication quality, comprehension of recommendations, service accessibility, treatment burden, and perceived barriers to adherence enables clinical teams to identify weaknesses across the preventive pathway and continuously optimize care delivery. Such feedback should assess the collective performance of the multidisciplinary care pathway rather than isolated clinical encounters, thereby reflecting shared responsibility across the health system (10, 12, 14).

Lifestyle modification and pharmacotherapy are complementary rather than mutually exclusive components of cardiovascular prevention. Pharmacological therapy should augment—rather than replace or unnecessarily delay—behavioral and environmental interventions when an individual’s cardiovascular risk or lipid burden indicates that lifestyle measures alone are unlikely to achieve recommended therapeutic targets (33, 34). Conversely, prescribing medication without addressing adherence, diet, physical activity, smoking status, and care continuity substantially limits the real-world effectiveness of otherwise evidence-based therapies (8, 9, 30).

From a health-system perspective, the success of non-pharmacological prevention should therefore be evaluated not only through short-term changes in body weight, blood pressure, glycemic indices, or lipid profiles, but also through long-term adherence, quality of life, patient experience, accessibility, equity, and cost-effectiveness. These dimensions ultimately determine whether an intervention can be implemented at scale and sustained beyond highly selected or particularly motivated patient populations (9, 10, 12, 16).

### 4.2 Lipid targets and treatment intensity

In clinical practice, therapeutic lipid targets are determined by total cardiovascular risk. A higher cardiovascular risk profile requires lower LDL-C targets and greater relative reductions from baseline (33, 34). Additional therapeutic targets include non-HDL-C and apolipoprotein B (ApoB), which provide complementary insight into the total burden of circulating atherogenic lipoproteins, particularly in patients with hypertriglyceridemia, diabetes, obesity, metabolic syndrome, or discordance between cholesterol concentration and atherogenic particle number (31, 34, 40, 43).

This paradigm reflects a broader shift from evaluating isolated lipid concentrations toward assessing total ApoB-containing particle burden and cumulative duration of arterial exposure (40, 43). Nevertheless, LDL-C remains the primary therapeutic target because of extensive randomized controlled trial evidence, standardized measurement methods, and well-established treatment thresholds. ApoB and non-HDL-C should therefore be regarded as complementary markers that refine risk assessment and therapeutic monitoring rather than as universal replacements for LDL-C (31, 34, 40, 43).

Lipid-lowering pharmacotherapy has traditionally been intensified through a stepwise strategy, beginning with maximally tolerated statin therapy, followed by the addition of ezetimibe and, when necessary, PCSK9-targeted therapies. Treatment selection should be guided by baseline cardiovascular risk, pretreatment and achieved lipid levels, tolerability, and the magnitude of additional LDL-C reduction required to reach the therapeutic target (33, 34).

Increasingly, however, contemporary clinical guidance supports earlier use of combination lipid-lowering therapy in patients who are unlikely to achieve recommended LDL-C targets with statin monotherapy alone. In such individuals, unnecessarily delaying treatment intensification prolongs exposure of the arterial wall to residual atherogenic lipoprotein burden and may diminish the lifetime cardiovascular benefit achievable through earlier and more effective lipid lowering (34, 40).

Treatment intensity should therefore be aligned with baseline LDL-C concentration, overall cardiovascular risk, projected lifetime exposure, comorbidities, the initial therapeutic gap, and the probability of achieving recommended targets within a clinically meaningful timeframe (33, 34, 40). Treatment optimization should be proactive rather than exclusively reactive; repeated cycles of inadequate therapy unnecessarily prolong cumulative arterial exposure to atherogenic lipoproteins. Contemporary preventive cardiology therefore supports timely regimen optimization in patients with high lifetime cardiovascular risk, particularly when multiple cardiometabolic abnormalities coexist (4, 33, 34, 40).

Importantly, the clinical benefit of lipid-lowering therapy is closely related to both the magnitude of reduction in circulating ApoB-containing lipoproteins and the duration for which this reduction is maintained (40). Earlier and sustained reduction of the atherogenic lipoprotein burden limits cumulative lifetime arterial exposure and thereby reduces long-term ASCVD risk (40). This exposure-response relationship underscores the importance of timely treatment initiation, adequate dose titration, achievement of therapeutic targets, and long-term persistence with effective therapy rather than delayed escalation.

However, the availability of potent lipid-lowering agents does not automatically translate into population-level cardiovascular risk reduction. Real-world studies consistently demonstrate substantial gaps between guideline recommendations and achieved LDL-C targets. These gaps reflect multiple factors, including underprescribing, delayed treatment intensification, true or perceived statin intolerance, financial and reimbursement constraints, fragmented follow-up, and non-adherence. The DA VINCI study demonstrated that a substantial proportion of European patients—including those at high and very high cardiovascular risk—fail to achieve recommended lipid targets despite the availability of effective lipid-lowering strategies (49, 50).

Adherence remains a pivotal determinant of therapeutic success. Apparent pharmacological resistance may reflect inconsistent medication intake, premature discontinuation, complex dosing regimens, adverse effects, inadequate communication, or limited access to treatment rather than true biological non-responsiveness. Simplifying therapeutic regimens through fixed-dose combinations, reducing pill burden where feasible, providing structured follow-up, and involving pharmacists and nurses in longitudinal management can improve treatment persistence and target attainment (9, 16, 30).

Consequently, medication adherence should be evaluated as a measurable clinical outcome rather than regarded solely as an individual patient responsibility. Health systems should systematically identify barriers to adherence—including regimen complexity, adverse events, financial strain, communication gaps, and structural obstacles—and implement targeted solutions before preventable treatment failure translates into adverse cardiovascular outcomes (8–10, 12, 16, 30).

Digital health tools can further support lipid management by identifying undertreated patients, generating automated clinical reminders, monitoring therapeutic goal attainment, and providing structured feedback to healthcare professionals. Quality-improvement initiatives integrating electronic health records, clinical registries, digital dashboards, and performance feedback may strengthen adherence to guideline-directed therapy; however, scalable implementation requires high-quality data, system interoperability, clinician engagement, appropriate governance, and equitable digital access (9, 10).

Digital innovations should therefore function as enabling mechanisms rather than standalone solutions. Clinical decision-support systems, remote monitoring, and artificial intelligence can strengthen preventive care only when they are embedded within coordinated clinical workflows, implemented by appropriately trained multidisciplinary teams, and made accessible across diverse socioeconomic groups (9, 10, 12).

Economic implications must also be considered. Although intensive lipid-lowering therapy may increase short-term pharmaceutical expenditure, preventing myocardial infarction, stroke, disability, hospitalization, and premature mortality can generate substantial long-term health and economic benefits. Health-economic evaluations should therefore extend beyond drug acquisition costs to include averted cardiovascular events, preserved work capacity and productivity, reduced informal care requirements, quality-adjusted life-years (QALYs), and the operational costs of adherence-support and care-coordination programs (12, 16).

Ultimately, successful lipid management requires integration of biological evidence with implementation science. Validated therapeutic targets, effective pharmacological interventions, and informative biomarkers must be supported by accessible healthcare delivery, timely treatment intensification, shared decision-making, sustained adherence support, periodic reassessment, and effective communication across primary care, laboratory medicine, cardiology, endocrinology, pharmacy, and patient networks. Only such an integrated framework can translate the efficacy demonstrated in clinical trials into durable real-world outcomes. More broadly, successful cardiometabolic prevention depends on the convergence of clinical evidence, coordinated healthcare delivery, multidisciplinary collaboration, continuous quality improvement, patient-reported feedback, equitable access to care, and shared accountability across the health system (8–10, 12, 14).

## 5 Cardiovascular risk as a lifelong process

Atherosclerosis originates decades before its first clinical manifestation, evolving silently as a progressive subclinical process driven by cumulative arterial exposure to atherogenic lipoproteins and other cardiometabolic risk factors (24, 40, 42). At the cellular and tissue levels, this chronic process involves the progressive retention and modification of apolipoprotein B–containing lipoproteins within the arterial intima, initiating and sustaining inflammatory responses that promote plaque development, progression, and maturation (24, 29, 40, 43).

Importantly, the biological consequences of cardiovascular risk factors depend not only on their magnitude but also on the duration of exposure. Moderately elevated LDL-C, blood pressure, body weight, or glycemia sustained over several decades can generate a substantial cumulative cardiometabolic burden, even when conventional short-term risk models classify an individual as being at low risk. This discrepancy is particularly relevant in younger adults, in whom 10-year risk algorithms may underestimate the long-term consequences of persistent metabolic abnormalities because chronological age exerts a strong influence on short-term absolute risk estimates (15, 27, 33, 40).

Accordingly, contemporary cardiovascular prevention is increasingly based on four complementary principles: initiating appropriate interventions early in life, maintaining effective prevention across the lifespan, integrating lifestyle measures with evidence-based pharmacotherapy when indicated, and tailoring intervention intensity to total cardiovascular risk (11, 14, 33, 34). This approach operationalizes the concept of life-course risk by recognizing that exposure to atherogenic and cardiometabolic drivers accumulates from early adulthood and that the timing of risk-factor modification influences the total lifetime burden of disease (40, 42). From a clinical perspective, risk assessment should therefore not be deferred until symptomatic cardiovascular disease emerges, but should occur sufficiently early to identify modifiable risk while meaningful opportunities for disease prevention remain (11, 33, 40).

A life-course paradigm also requires iterative risk reassessment rather than reliance on a single baseline estimate. Cardiovascular risk evolves dynamically with age, changes in body composition, major reproductive transitions such as pregnancy and menopause, the development of diabetes or chronic kidney disease, exposure to psychosocial and environmental stressors, treatment persistence, and changing socioeconomic circumstances. Consequently, a risk estimate obtained at a single point in time may become clinically outdated and should be interpreted within a dynamic preventive care model rather than as a permanent risk assignment (9, 14, 15, 33).

The greatest opportunity to modify the natural history of cardiometabolic disease occurs while individuals remain asymptomatic and before advanced vascular or end-organ damage has developed. Once overt disease emerges, clinical complexity, disability, healthcare utilization, and economic costs increase substantially (10–12, 35). Successful prevention should therefore be evaluated not only by reductions in recurrent events among individuals with established disease, but also by the capacity of health systems to identify high-risk individuals early, maintain long-term engagement, and interrupt disease progression across the life course (8, 9, 11, 12, 14).

This strategy does not imply universal or indiscriminate pharmacological treatment of all young or asymptomatic individuals. Rather, it supports the timely and targeted identification of individuals with inherited or persistently elevated risk, including those with severe dyslipidemia, familial hypercholesterolemia, markedly elevated lipoprotein(a), obesity, hypertension, diabetes, or evidence of early subclinical atherosclerosis. The intensity of intervention must remain proportional to absolute and lifetime risk, anticipated clinical benefit, treatment burden, patient preferences, and the feasibility of maintaining long-term safety and adherence (15, 30, 32–34).

Despite major advances in preventive cardiology, a substantial evidence-to-practice gap persists. Cardiometabolic diseases remain leading global causes of morbidity and mortality partly because effective preventive interventions are frequently initiated too late, implemented incompletely, or applied inconsistently across health systems (3, 8–12, 17). From a life-course perspective, these delays are particularly consequential because each period of untreated or insufficiently treated risk contributes to cumulative biological exposure.

Delayed prevention reflects failures at multiple points along the care pathway. Cardiovascular risk may remain undetected because of irregular screening, isolated interpretation of laboratory parameters, incomplete family histories, or excessive reliance on short-term risk estimates that may classify younger individuals as low risk despite substantial lifetime exposure. Even when risk is identified, clinical action may be delayed by therapeutic inertia, fragmented referral pathways, time-constrained consultations, inadequate follow-up, restrictive reimbursement policies, and ineffective communication between primary and specialist care (8–10, 12, 15, 27). These barriers are cumulative: multiple relatively small failures across the diagnostic and therapeutic pathway can collectively result in missed opportunities for early prevention (8–10).

Preventive strategies therefore achieve their greatest potential when initiated at an appropriate stage, applied consistently, and sustained before subclinical disease progresses to irreversible vascular damage. Prevention should be conceptualized not as an isolated clinical encounter or a single prescription, but as a continuous and adaptive process encompassing serial risk reassessment, therapeutic titration, behavioral support, and coordinated longitudinal follow-up, reinforced by system-wide quality-improvement mechanisms (9, 11, 14, 33, 40).

Delivering such continuity requires multidisciplinary collaboration among patients, primary care clinicians, laboratory specialists, nurses, pharmacists, dietitians, cardiologists, endocrinologists, rehabilitation professionals, and public health services. Equally important is the longitudinal integration of clinical findings, laboratory measurements, medication history, behavioral patterns, environmental exposures, socioeconomic circumstances, and patient-reported information into a coherent cardiometabolic profile. Without such integration, individual abnormalities may be addressed in isolation while their cumulative biological and clinical significance remains unrecognized (9, 10, 12, 14).

Active patient engagement is integral to this life-course model. Long-term risk reduction is difficult to sustain when recommendations are delivered without shared decision-making, transparent communication of absolute and lifetime risk, or consideration of treatment burden and individual preferences. Patient-reported outcome measures (PROMs) and patient-reported experience measures (PREMs) provide complementary information regarding symptom burden, functional status, treatment tolerability, continuity of care, and real-world barriers to adherence. Systematic collection of this information can identify vulnerabilities within preventive pathways that conventional laboratory targets or hospitalization metrics alone cannot capture (9, 12, 14). Evaluating patient feedback across the disciplines involved in care can further support continuous quality improvement and shared accountability between patients and healthcare teams (9, 12, 14).

Digital health solutions may further strengthen lifelong prevention by facilitating repeated risk assessment, electronic decision support, medication reminders, remote monitoring, and communication across different levels of care. Their effectiveness, however, depends on technical interoperability, data integrity, digital health literacy, clinician acceptance, data governance and privacy, and equitable access. Digitalization should therefore serve as an implementation accelerator for coordinated, person-centered preventive care rather than as a substitute for clinical continuity or multidisciplinary engagement (9, 10).

Early and sustained intervention also has important economic implications. Delayed prevention contributes to myocardial infarction, stroke, heart failure, disability, hospitalization, long-term polypharmacy, loss of productivity, and increased reliance on formal and informal caregiving. Although structured prevention requires upfront investment, health-economic evaluations should account for averted clinical events, preserved work capacity, reduced disability, gains in quality-adjusted life-years (QALYs), and lower long-term demand for high-cost tertiary care rather than focusing exclusively on short-term pharmaceutical or consultation expenditure (12, 16).

In conclusion, conceptualizing cardiovascular risk as a lifelong process provides the temporal framework required to connect atherosclerotic biology with effective prevention. The central issue is not simply whether an individual meets a treatment threshold at a particular clinical encounter, but how the magnitude, duration, and interaction of risk factors shape cumulative cardiovascular burden over time. Population-level benefit therefore depends on the capacity of health systems to identify clinically meaningful risk early, reassess it dynamically, initiate proportionate intervention, and sustain effective prevention throughout the life course (8, 10, 15, 33, 40). This life-course perspective provides the foundation for the subsequent consideration of how preventive knowledge can be translated into coordinated, measurable, equitable, and sustainable clinical practice.

## 6 Current limitations of contemporary cardiometabolic prevention and future research priorities

Despite major advances in cardiovascular biology, risk prediction, lipid-lowering pharmacotherapy, obesity management, diabetes care, and lifestyle medicine, cardiometabolic diseases remain the primary drivers of premature mortality worldwide (3, 8, 11, 17). This apparent paradox highlights a fundamental challenge in contemporary preventive cardiology: generating robust scientific evidence is insufficient unless that evidence can be translated into timely, coordinated, equitable, and sustainable clinical practice. The effectiveness of prevention is therefore increasingly determined by the capacity of health systems to bridge the evidence-to-practice gap (8, 10).

Implementation studies demonstrate that even well-established, effective, and cost-efficient preventive interventions frequently fail to reach the individuals most likely to benefit. Krumholz emphasized that advances in risk prediction and therapeutics continue to coexist with major implementation barriers, particularly in resource-limited, socioeconomically disadvantaged, and fragmented healthcare settings (8). Similarly, Dores et al. conceptualized cardiovascular prevention as a continuous pathway encompassing risk detection, stratification, targeted intervention, monitoring, and iterative reassessment, with opportunities for failure at each stage (10). These observations challenge the assumption that increasingly detailed clinical guidelines will, by themselves, improve population outcomes. Recommendations generate measurable health benefits only when they are incorporated into coherent care pathways, delivered by appropriately trained multidisciplinary teams, understood and accepted by patients, and evaluated using clinically meaningful quality indicators (9–12, 14).

### 6.1 Limitations of current cardiovascular risk prediction

Contemporary algorithms, including SCORE2 and SCORE2-OP, represent major advances in European cardiovascular risk estimation by incorporating fatal and non-fatal cardiovascular events, providing regional calibration, and supporting risk-proportional intervention (27, 28, 33). Nevertheless, important structural and practical limitations remain.

A clinically relevant limitation concerns how SCORE2 is operationalized in routine practice. Simplified paper-based risk charts approximate the underlying continuous SCORE2 equations by categorizing continuous variables into predefined intervals. Consequently, cardiovascular risk classification may differ depending on whether risk is estimated using printed charts or the continuous mathematical models implemented in digital calculators. Goławski et al. demonstrated that SCORE2 and SCORE2-OP paper charts can misclassify a substantial proportion of primary care patients compared with their underlying continuous models (38). Hageman et al. subsequently showed that the choice between chart-based and calculator-based implementation can materially influence model performance, treatment eligibility, and the estimated number of preventable cardiovascular events (39). Thus, the mode of SCORE2 implementation is not merely a technical consideration; it can directly influence clinical decision-making. Whenever feasible, validated digital calculators implementing the continuous SCORE2 equations should therefore be preferred, particularly when estimated risk lies close to age-specific treatment thresholds (38, 39). These findings demonstrate that the clinical performance of a risk model depends not only on its mathematical architecture but also on the fidelity with which it is implemented in practice (38, 39).

Beyond these implementation considerations, conventional cardiovascular risk models have several broader limitations:

- Restricted time horizons: Standard risk models generally estimate cardiovascular risk over a fixed period, most commonly 10 years. Because age is a major determinant of short-term absolute risk, younger individuals with severe dyslipidemia, inherited susceptibility, obesity, diabetes, prolonged exposure to atherogenic lipoproteins, or subclinical atherosclerosis may nevertheless be classified as having low short-term risk (15, 27, 33, 40, 42). This creates an important clinical paradox in which a deceptively low 10-year risk estimate may coexist with substantial lifetime cardiovascular risk.
- Incomplete representation of biological and environmental heterogeneity: Conventional calculators rely on a relatively limited set of established risk factors and therefore cannot fully capture the biological, genetic, behavioral, social, and environmental heterogeneity underlying cardiovascular disease. Family history of premature coronary artery disease provides a relevant example. As emphasized by Lan et al. (15), family history reflects a complex interaction among inherited genetic susceptibility, shared environmental exposures, and lifestyle patterns. Despite its established prognostic relevance, family history remains inconsistently defined, incompletely documented, and variably incorporated into routine cardiovascular risk assessment (15).
- Unresolved integration of novel biomarkers and imaging: The optimal integration of coronary artery calcium (CAC) scoring, vascular imaging, apolipoprotein B (ApoB), lipoprotein(a), high-sensitivity inflammatory biomarkers, genomic profiling, and polygenic risk scores (PRS) into routine clinical pathways remains incompletely standardized (10, 15, 30–33). Although these approaches may improve risk discrimination or reclassify selected individuals near therapeutic decision thresholds, incremental gains in statistical prediction do not necessarily translate into improved clinical outcomes.

A biomarker or imaging modality becomes clinically valuable when the additional information it provides meaningfully changes management, improves risk communication or adherence, or ultimately contributes to better patient outcomes. Future evaluation of emerging risk markers should therefore extend beyond C-statistics and reclassification indices to include clinical utility, patient acceptability, feasibility, equity, cost-effectiveness, and long-term effects on clinically meaningful outcomes. Cardiovascular risk assessment may consequently evolve from static, single-point estimates toward more dynamic and multimodal approaches integrating clinical, biomarker, imaging, behavioral, environmental, and patient-reported information (10, 15).

### 6.2 Precision prevention: opportunities and unresolved questions

Precision cardiovascular prevention seeks to complement conventional clinical assessment with genomic, imaging, advanced biomarker, behavioral, and environmental information. Such an approach may be particularly valuable for identifying high-risk individuals whose cumulative or biologically determined risk is insufficiently captured by conventional algorithms (10, 15).

Polygenic risk scores may identify inherited susceptibility before conventional risk factors or clinical cardiovascular disease become apparent. Advanced vascular imaging can identify subclinical atherosclerotic burden and potentially refine risk estimates in selected patients. Similarly, multimodal approaches may integrate laboratory findings, imaging, family history, medication exposure, lifestyle determinants, and patient preferences to support more individualized preventive decision-making (15, 30, 33).

However, the potential of precision prevention should be distinguished from demonstrated clinical utility. Several major questions must be resolved before widespread implementation:

- Incremental value and equity gaps: The additional predictive value of PRS beyond established clinical risk tools is often modest, while performance may differ substantially across ancestral populations. Premature or uncritical implementation could therefore exacerbate existing health inequalities, particularly while genomic reference datasets remain disproportionately derived from populations of European ancestry (15).
- Cost, radiation, and infrastructure barriers: Imaging modalities such as CAC scoring involve financial costs, ionizing radiation, and infrastructure requirements that may restrict accessibility, particularly in resource-constrained health systems. These limitations favor selective, clinically justified imaging rather than indiscriminate population screening (15, 30, 33).
- Impact on clinically meaningful outcomes: It remains uncertain whether precision profiling improves hard cardiovascular outcomes rather than merely refining predicted risk. Prospective studies should determine whether incorporating genomics, CAC, or novel biomarkers into clinical pathways leads to meaningful changes in treatment, sustained behavioral modification, improved medication persistence, and ultimately fewer cardiovascular events (10, 15).
- Economic viability: Emerging diagnostic technologies must demonstrate that their incremental clinical value justifies their financial and organizational burden. Precision prevention should not be equated with performing more tests; rather, it should involve the selective use of additional diagnostic information when that information meaningfully improves decision-making, patient outcomes, or healthcare resource allocation (10, 12, 15).

Accordingly, the future of precision prevention depends less on the number of available biomarkers or technologies than on evidence that their integration into clinical pathways produces actionable information and measurable benefit.

### 6.3 The evidence-to-practice gap

The real-world effectiveness of cardiometabolic prevention is constrained by an interconnected network of barriers operating across health-system, clinician, and patient levels. These barriers rarely act independently. Instead, they accumulate throughout the preventive pathway, creating a sequence of missed opportunities that can delay risk recognition, postpone intervention, impair treatment persistence, and reduce the effectiveness of otherwise evidence-based therapies (8–10, 12).

At the health-system level, prevention is undermined by fragmented care pathways, poor coordination between primary and specialist services, inadequate infrastructure, misaligned reimbursement models, and insufficient institutional support for long-term preventive strategies (8–10, 12). Health systems that remain predominantly oriented toward acute disease management may fail to provide the care continuity required for proactive risk identification, longitudinal monitoring, and sustained intervention, despite the substantial clinical and economic burden associated with delayed prevention (10–12, 16).

At the clinician level, therapeutic inertia represents an important barrier when treatment is not initiated or intensified despite persistent failure to achieve appropriate risk-factor targets. Its causes are multifactorial and include time-constrained consultations, competing clinical priorities, underestimation of lifetime risk, limited use of validated decision-support tools, unfamiliarity with evolving recommendations, and concerns regarding adverse effects or treatment complexity (8–10). Similarly, lifestyle counseling is unlikely to produce sustained behavioral change when reduced to brief, generic recommendations. Effective behavioral intervention requires individualized goals, longitudinal reinforcement, and access to appropriate multidisciplinary support (9, 10, 14).

At the patient level, adherence cannot be understood solely as an expression of individual motivation. Medication persistence and sustained behavioral change are influenced by health literacy, treatment burden, adverse-effect concerns, financial constraints, psychosocial stress, competing priorities, cultural factors, and accessibility of care (9, 10, 12, 22). The surrounding environment further influences the feasibility of preventive recommendations, particularly where physical inactivity, highly processed foods, inadequate sleep, or chronic stress are structurally reinforced (1, 12, 21, 52).

These barriers form an interconnected causal pathway. Fragmented services may impair follow-up and communication; inadequate communication can reduce patient understanding and adherence; poor adherence may be interpreted as pharmacological treatment failure; and delayed reassessment or treatment intensification can prolong exposure to modifiable cardiovascular risk. Conversely, clinicians cannot consistently deliver high-value preventive care when consultation time, multidisciplinary resources, reimbursement structures, or longitudinal follow-up mechanisms are inadequate. The evidence-to-practice gap should therefore be understood as a systems problem rather than the consequence of isolated clinician or patient behavior (9, 10, 12).

Implementation science is consequently an essential component of contemporary cardiovascular prevention. Preventive success should be evaluated not only through isolated process measures, but also through patient-centered outcomes, care continuity, treatment persistence, quality of life, cardiovascular events, resource utilization, operational efficiency, equity, and long-term sustainability (8–10, 12, 16).

### 6.4 Patient-reported feedback as a measure of integrated care quality

Evaluation of the patient’s experience across the preventive pathway represents an important but still underdeveloped dimension of integrated cardiometabolic care. Laboratory targets, prescribing rates, and hospital utilization provide essential information regarding clinical performance, but they do not establish whether care was experienced as accessible, understandable, coordinated, respectful, and coherent (9, 10, 12).

Integrated preventive pathways should therefore incorporate structured longitudinal patient feedback at clinically meaningful stages of care. Evaluation should encompass the entire patient journey across primary care, laboratory diagnostics, nursing, dietetic and behavioral support, pharmacy, cardiology, endocrinology, hepatology, rehabilitation, and public health services (9, 12, 14).

Because failures in cardiometabolic prevention frequently occur during transitions between providers or levels of care, pathway-level assessment may provide greater quality-improvement value than isolated measures of satisfaction with individual encounters (9, 10, 12, 14). Relevant domains include:

- Timeliness of diagnostic evaluation and specialist referral;
- Clarity and consistency of communication across disciplines;
- Continuity between primary and specialist care;
- Accessibility of diagnostic testing and prescribed treatment;
- Patient participation in shared decision-making;
- Understanding of individual cardiovascular risk and treatment goals;
- Consistency of lifestyle and pharmacological recommendations;
- Reliability of follow-up and monitoring;
- Consideration of patient preferences, health literacy, and financial circumstances; and
- The patient’s perception of coordination across the multidisciplinary team.

Patient-Reported Experience Measures (PREMs) should, where feasible, be interpreted alongside Patient-Reported Outcome Measures (PROMs), clinical outcomes, medication adherence indicators, and healthcare utilization data. Such integration allows health systems to evaluate not merely whether guideline-directed treatment was prescribed, but whether the organization of care was sufficiently coherent to support its sustained implementation (9, 12, 14).

Importantly, patient feedback has limited value unless incorporated into a closed-loop quality-improvement process. Data collection should therefore be followed by systematic analysis, multidisciplinary review, identification of operational barriers, implementation of corrective actions, and subsequent reassessment. Within a value-based framework, patient-reported information can thus complement hard clinical outcomes by identifying weaknesses in communication, accessibility, coordination, and care continuity that conventional biomedical metrics may fail to capture (10, 12, 14).

### 6.5 Digital prevention and artificial intelligence

Digital health technologies may facilitate several components of longitudinal cardiovascular prevention, including physiological monitoring, repeated risk assessment, medication adherence support, remote consultation, and personalized follow-up. Wearable devices, mobile applications, telemedicine, digital referral networks, and artificial intelligence (AI)-supported clinical decision tools may also help improve communication and care continuity between primary and specialist care (10, 14).

Artificial intelligence should be regarded as a decision-support technology rather than a substitute for clinical judgment. Its potential clinical value lies in supporting the integration of complex, high-dimensional datasets that may be difficult to synthesize consistently in routine practice, thereby assisting clinicians in identifying high-risk phenotypes, refining individualized management pathways, and maintaining care continuity across fragmented care settings (10, 14, 15).

Digital platforms may also facilitate longitudinal collection of PREMs and PROMs. Brief electronic surveys administered after consultations, diagnostic procedures, or treatment modifications could provide timely information regarding accessibility, communication, treatment burden, and patient understanding. When appropriately governed and analyzed, aggregated patient-reported data may help identify recurrent weaknesses at specific points in the care pathway (10, 12, 14).

Nevertheless, the real-world clinical effectiveness of digital prevention remains incompletely established. Many digital interventions have been evaluated over relatively short follow-up periods, in selected populations, and using surrogate rather than hard cardiovascular endpoints. Evidence regarding sustained event reduction, cost-effectiveness, interoperability, data security, and effective integration into routine clinical workflows remains limited (10).

Digital technologies also have important implications for health equity. Although they may expand access for geographically isolated or underserved populations, they can simultaneously widen the digital divide. Individuals with limited access to devices, inadequate connectivity, low digital literacy, or language barriers may be disproportionately excluded despite often carrying substantial baseline cardiovascular risk (9, 10, 12).

Future evaluations should therefore assess digital interventions not only in terms of technical performance, but also according to usability, accessibility, equity, patient burden, workflow compatibility, cost-effectiveness, and unintended consequences. Digital feedback systems should remain simple, adaptable to different levels of health and digital literacy, and complemented by non-digital alternatives where necessary (9, 10, 12).

Similarly, AI-based algorithms require rigorous external validation across diverse demographic and clinical populations. Transparent governance frameworks are essential to address algorithmic bias, data privacy, explainability, accountability, and clinical responsibility. AI should therefore augment—rather than replace—clinical reasoning, multidisciplinary collaboration, and person-centered care (10, 12, 15).

### 6.6 Priorities for future research and implementation

Future research in cardiometabolic prevention should extend beyond the development of increasingly sophisticated risk algorithms and focus on whether new strategies improve clinically meaningful outcomes when implemented in real-world health systems (8, 10, 15). Several priorities emerge:

- Clinical utility of precision modalities: Prospective studies should determine whether incorporating genomic profiling, coronary artery calcium scoring, ApoB, lipoprotein(a), inflammatory biomarkers, environmental measures, and social determinants of health into routine care produces clinically meaningful changes in management and ultimately reduces hard cardiovascular events (10, 15, 30, 33).
- Life-course implementation models: Research should evaluate practical strategies for identifying and managing younger individuals who have low short-term but potentially high lifetime cardiovascular risk, including those with severe dyslipidemia, familial hypercholesterolemia, obesity, diabetes, or early subclinical atherosclerosis (15, 27, 33, 40, 49). Particular attention should be given to determining when earlier intervention provides sufficient long-term benefit to justify treatment burden and sustained healthcare resource use.
- Scalable integrated care pathways: Comparative implementation studies should determine whether coordinated models connecting primary care, laboratory medicine, nursing, cardiology, endocrinology, hepatology, nutrition, behavioral psychology, pharmacy, rehabilitation, and public health improve care continuity, adherence, therapeutic target attainment, PROMs, PREMs, quality of life, healthcare utilization, and clinically meaningful outcomes compared with conventional fragmented care (9, 10, 12, 14).
- Pathway-level patient feedback systems: Research should establish which PREM and PROM instruments are sufficiently brief, reliable, scalable, and actionable for longitudinal preventive care and whether systematic responses to patient feedback improve team coordination, trust, treatment persistence, care continuity, and clinical outcomes (9, 10, 12, 14).
- Equity and cost-effectiveness: Health equity and economic value should be incorporated as central outcomes of preventive implementation research. Diagnostic or therapeutic innovations with high technical performance but limited accessibility may generate little population-level benefit. Conversely, relatively simple interventions embedded within primary care or community settings may yield substantial health gains when they are affordable, scalable, and broadly accessible (9, 10, 12, 16, 22).
- Broader determinants of cardiometabolic health: Research must also address structural determinants of cardiometabolic risk, including food environments, socioeconomic inequality, educational disparities, occupational exposures, air pollution, and commercial determinants of health. Individual clinical counseling alone cannot fully compensate for environments that systematically impede sustained healthy behavior (1, 12, 21, 22, 35).

In summary, contemporary cardiometabolic prevention is constrained not simply by limitations in scientific knowledge, but by the difficulty of translating established evidence into coordinated, sustained, and equitable clinical action. Closing this evidence-to-practice gap requires rigorous implementation research, appropriately targeted precision approaches, integrated multidisciplinary care, meaningful patient-reported evaluation, responsible use of digital technologies, and continuous assessment of clinical effectiveness, equity, and economic sustainability. These principles are illustrated in the following clinical vignette, which demonstrates how longitudinal synthesis of routinely available clinical and laboratory information can reveal actionable cardiometabolic risk before overt disease becomes clinically apparent (8, 10, 15).

## 7 An educational clinical vignette: integrating routine data into lifelong cardiometabolic prevention

The preceding sections have demonstrated that contemporary cardiometabolic prevention extends beyond the identification of isolated biomarkers or the achievement of discrete therapeutic targets. Its real-world effectiveness depends on the longitudinal integration of biological, clinical, laboratory, behavioral, and organizational information across the life course. The following educational vignette illustrates how these dimensions converge in routine clinical practice, where opportunities for prevention often arise not from advanced diagnostic technologies, but from the systematic interpretation of routinely collected data (8–10, 12, 33).

This vignette is presented as an educational and translational example rather than as a formal case report or a source of generalizable clinical evidence. Its purpose is to demonstrate how the principal concepts discussed throughout this review—including cumulative exposure, limitations of short-term risk assessment, primary and secondary causes of dyslipidemia, diagnostic fragmentation, patient engagement, and coordinated care—intersect in the assessment of an apparently healthy young adult (8, 9, 33). It is intended to illustrate clinical reasoning and a proportionate diagnostic approach rather than to prescribe a specific diagnostic or therapeutic protocol.

The educational objectives of this vignette are to:

1. Distinguish low short-term cardiovascular risk from potentially elevated lifetime risk (15, 27, 33, 40).
2. Demonstrate the value of longitudinal rather than isolated cross-sectional laboratory interpretation (9, 10, 33).
3. Identify clinical triggers for evaluating primary and secondary causes of dyslipidemia (33, 45–47).
4. Illustrate the integration of primary care, laboratory medicine, and specialist services within a stepwise preventive pathway (9, 10, 14).
5. Demonstrate how integrated interpretation of routine data can enhance care continuity and support shared decision-making across healthcare settings (9, 10, 12, 14).

### 7.1 Initial presentation: low perceived risk despite persistent abnormalities

A 32-year-old woman presented to her primary care physician for a routine health assessment prompted by persistently abnormal lipid profiles documented over several years. She reported no clinically significant symptoms and perceived herself to be in good overall health. She maintained an active lifestyle, exercised regularly, did not smoke, and consumed alcohol only occasionally.

A detailed dietary history nevertheless identified several potentially unfavorable environmental and qualitative dietary factors, including frequent consumption of ultra-processed and ready-to-eat foods, relatively high exposure to saturated fats associated with dining out, and limited control over ingredient quality in commercial and institutional catering (1, 21, 52). These factors may contribute to excess energy and saturated fat intake even in individuals who perceive their overall lifestyle as healthy (1, 9, 21).

An important limitation of her previous management was the episodic interpretation of laboratory findings. Earlier lipid measurements had largely been considered as individual results rather than as part of a longitudinal trajectory incorporating cumulative exposure, treatment response, and the broader clinical context (9, 10, 15).

Her body mass index (BMI) was approximately 25.0 kg/m^2^, placing her at the threshold between normal weight and overweight. During the preceding years, she had experienced an insidious weight gain of several kilograms without intentional lifestyle changes. Although neither finding was independently diagnostic, together with the persistent lipid abnormalities they warranted longitudinal metabolic assessment.

Because of sustained elevations in total cholesterol and LDL-C, the patient had previously been referred for cardiological evaluation. Approximately two years earlier, atorvastatin had been initiated at a standard dose in the context of persistent LDL-C elevation and a positive family history of metabolic risk (33, 34, 40).

Despite reported adherence, no clinically meaningful reduction in lipid concentrations had been documented. Rather than being interpreted immediately as evidence of pharmacological resistance or secondary endocrine disease, an unexpectedly limited therapeutic response should prompt systematic verification of the prescribed dose and statin intensity, treatment duration, actual medication exposure, adherence, potential drug interactions, and the expected magnitude of LDL-C reduction (33, 34, 45–47).

Her medical history included bronchial asthma diagnosed at 14 years of age, currently asymptomatic and requiring no maintenance therapy. Her mother and maternal grandmother had a history of thyroid disease. This family history did not establish an endocrine etiology of the patient’s dyslipidemia, but increased the relevance of thyroid function within a proportionate assessment of potential secondary contributors (15, 33, 46, 47).

Taken together, persistent dyslipidemia, gradual weight gain, an apparently limited response to lipid-lowering therapy, and relevant family history justified a structured reassessment rather than empirical treatment escalation. The principal clinical task was to confirm the lipid phenotype, verify treatment exposure, evaluate primary and plausible secondary causes of dyslipidemia, and interpret short-term cardiovascular risk within the context of cumulative lifetime exposure (15, 27, 33, 34, 40, 45–47).

This pattern illustrates a central principle of preventive medicine: clinically meaningful risk may emerge from the longitudinal relationship among individually modest or apparently unrelated findings rather than from a single diagnostic threshold (9, 10, 33) (see Tables 1–4).

**Table 1:** Complete blood count results at presentation.

Parameter	Result	Unit	Reference range	Comments
Leukocytes	8.13	10^3^/μL	3.98–10.04	
Hemoglobin (Hb)	13.20	g/dL	11.20–15.70	
Red blood cells (RBC)	4.23	10^6^/μL	3.93–5.22	
Hematocrit (Hct)	40.3	%	34.1–44.9	
Mean corpuscular volume (MCV)	95.3	fL	79.4–94.8	H
Mean corpuscular hemoglobin (MCH)	31.3	pg	25.6–32.2	
Mean corpuscular hemoglobin concentration (MCHC)	32.8	g/dL	32.2–35.5	
Platelets (PLT)	309.0	10^3^/μL	150.0–400.0	
Red cell distribution width-standard deviation (RDW-SD)	45.0	fL	36.4–46.3	
Red cell distribution width-coefficient of variation (RDW-CV)	12.8	%	11.7–14.4	
Platelet distribution width (PDW)	15.9	fL	10.0–17.0	
Mean platelet volume (MPV)	10.0	fL	9.4–12.5	
Platelet large cell ratio (P-LCR)	29.5	%	19.1–46.6	
Plateletcrit (PCT)	0.3	%	0.2–0.4	
Neutrophils (absolute count)	3.37	10^3^/μL	2.00–7.00	
Lymphocytes (absolute count)	3.50	10^3^/μL	1.00–3.00	H
Monocytes	0.71	10^3^/μL	0.20–1.00	
Eosinophils	0.51	10^3^/μL	0.02–0.50	H
Basophils	0.04	10^3^/μL	0.02–0.10	
Neutrophils (%)	41.5	%	40.0–80.0	
Lymphocytes (%)	43.0	%	20.0–40.0	H
Monocytes (%)	8.7	%	2.0–10.0	
Eosinophils (%)	6.3	%	1.0–6.0	H

H, high.

**Table 2:** Liver function tests—results.

Parameter	Result	Unit	Reference range	Comments
Aspartate aminotransferase (AST)	25.4	U/L	5.0–34.0	
Alanine aminotransferase (ALT)	57.1	U/L	0.0–55.0	H
Total bilirubin	0.98	mg/dL	0.20–1.20	
Alkaline phosphatase (ALP)	44.1	U/L	40.0–150.0	
Gamma-glutamyl transferase (GGT)	20.7	U/L	9.0–36.0	

H, high.

**Table 3:** Lipid profile—results.

Parameter	Result	Unit	Reference range/risk-adjusted therapeutic targets	Comments
Total cholesterol (TC)	283.5	mg/dL	115.0–190.0	H
High-density lipoprotein cholesterol (HDL-C)	79.9	mg/dL	Recommended: > 45 mg/dL	
Non–high-density lipoprotein cholesterol (non-HDL-C)	203.6	mg/dL	Recommended values: < 130 mg/dL–moderate risk < 100 mg/dL–high risk < 85 mg/dL–very high risk < 70 mg/dL–extreme risk	
Low-density lipoprotein cholesterol (LDL-C)	180.1	mg/dL	Recommended values: < 115 mg/dL–low risk < 100 mg/dL–moderate risk < 70 mg/dL–high risk < 55 mg/dL–very high risk < 40 mg/dL–extreme risk	
Triglycerides (TG)	117.4	mg/dL	Fasting: <100 mg/dL Non-fasting: <125/dL	

H, high.

**Table 4:** TSH and inflammatory parameters—results.

Parameter	Result	Unit	Reference range
C-reactive protein (CRP), quantitative	1.98	mg/L	0.00–5.00
Erythrocyte sedimentation rate (ESR)	13	mm/h	2–15
Thyroid-stimulating hormone (TSH)	3.742	μIU/ml	0.350–4.940

### 7.2 Complete blood count: distinguishing incidental findings from actionable risk

The complete blood count (CBC) illustrates an important principle of integrated laboratory interpretation: not every laboratory abnormality represents cardiometabolic pathology, and minor deviations should be evaluated according to their magnitude, persistence, and clinical context.

The patient’s CBC showed mild absolute and relative lymphocytosis, slight eosinophilia, and a borderline-high mean corpuscular volume, with a normal total leukocyte count. Given her history of bronchial asthma, an allergic or atopic background provides a plausible explanation for the mild eosinophilia. Minor leukocyte abnormalities may also reflect transient reactive states or analytical variation and can therefore be reassessed by repeat testing or, when clinically appropriate, manual differential examination.

Although chronic low-grade inflammation contributes to obesity, insulin resistance, dyslipidemia, and atherogenesis (4, 5, 24), nonspecific findings such as mild lymphocytosis or eosinophilia should not be interpreted as evidence of vascular inflammation without appropriate clinical support. In this setting, a proportionate approach consists of follow-up testing and consideration of allergic, infectious, medication-related, or other clinically plausible explanations.

The educational relevance of the CBC therefore lies not in assigning these minor abnormalities to a unified cardiometabolic mechanism, but in demonstrating the importance of distinguishing incidental findings from reproducible and actionable indicators of risk (10, 33).

### 7.3 Liver enzymes as early markers of metabolic disturbances

The patient’s liver function tests demonstrated a mild, isolated elevation of alanine aminotransferase (ALT), while aspartate aminotransferase (AST), gamma-glutamyl transferase (GGT), alkaline phosphatase (ALP), and total bilirubin remained within their respective reference ranges. This pattern is compatible with mild hepatocellular stress but is inherently nonspecific and does not, in isolation, establish clinically significant liver disease, cholestasis, or hepatic functional impairment (6, 45).

In the context of persistent dyslipidemia and gradual weight gain, metabolic dysfunction-associated steatotic liver disease (MASLD) may reasonably be considered within the differential diagnosis. However, an isolated minimally elevated ALT concentration is insufficient to establish MASLD, and its clinical significance derives from integration with anthropometric, metabolic, clinical, and, when indicated, imaging findings (4, 6, 45, 53).

Current guidance supports a stepwise approach in which modest aminotransferase abnormalities are first confirmed and interpreted after consideration of transient confounding factors, including recent alcohol consumption, strenuous exercise, intercurrent illness, and medication exposure (6, 45, 53). Persistent abnormalities should prompt reassessment of metabolic risk factors and further investigation according to the overall clinical probability of liver disease.

Accordingly, the appropriate response in this vignette is not immediate extensive hepatological investigation, but repeat biochemical testing, review of potential confounders, assessment of glycemic and anthropometric parameters, and consideration of hepatic imaging if the abnormality persists or additional risk factors emerge (6, 45, 53).

This finding illustrates how a subtle laboratory abnormality may become clinically relevant when interpreted longitudinally, while also emphasizing the need to avoid overdiagnosis based on a single borderline result (4, 6, 9, 10, 33).

### 7.4 Lipid profile: real risk in a young individual

#### 7.4.1 Interpretation of the lipid profile in the context of atherosclerotic cardiovascular risk

The most clinically significant laboratory abnormality in this vignette was the lipid profile. Total cholesterol was 283.5 mg/dL, LDL-C 180.1 mg/dL, and non-HDL-C 203.6 mg/dL, indicating a substantial burden of circulating atherogenic lipoproteins (34, 40, 42, 43). Their clinical relevance is strengthened by persistence over time rather than by a single measurement alone.

Current European guidance emphasizes that marked and persistent LDL-C elevation warrants assessment in the context of overall cardiovascular risk and evaluation for potentially inherited or secondary causes of dyslipidemia (33, 34).

#### 7.4.2 Cardiovascular risk assessment according to SCORE2

SCORE2 and SCORE2-OP are recommended European tools for estimating 10-year cardiovascular risk in individuals without established cardiovascular disease (27, 28, 33). However, short-term risk estimates are strongly influenced by age and should not be interpreted as comprehensive measures of lifetime biological risk.

At 32 years of age, this patient would be expected to have a low short-term absolute cardiovascular risk in the absence of other major risk factors. Such classification does not negate the potential significance of prolonged exposure to markedly elevated LDL-C. In younger adults, clinical assessment should therefore integrate the magnitude and duration of lipid exposure, family history, potential familial hypercholesterolemia, secondary causes, and projected lifetime risk rather than relying exclusively on a 10-year risk estimate (15, 27, 33, 40).

This distinction is particularly relevant because cumulative exposure to ApoB-containing lipoproteins is a major determinant of atherosclerotic burden (15, 40). Cardiovascular risk algorithms should therefore inform, rather than replace, individualized clinical judgment (33, 38, 39).

#### 7.4.3 Non-HDL cholesterol and ApoB-containing particle burden

The patient’s non-HDL-C concentration was 203.6 mg/dL, substantially above recommended risk-adjusted targets. Non-HDL-C captures cholesterol contained in the major ApoB-containing atherogenic lipoprotein classes and provides a clinically accessible complementary measure of atherogenic burden, particularly when ApoB itself is not measured (34, 42, 43).

Although the patient’s HDL-C concentration was relatively high (79.9 mg/dL), this should not be interpreted as neutralizing the risk associated with persistent elevation of LDL-C and non-HDL-C. Contemporary evidence does not support using high HDL-C as a counterweight to sustained exposure to ApoB-containing particles, nor is HDL-C considered a direct therapeutic target (24, 33, 34, 40).

The key clinical issue is therefore not a single lipid measurement, but the cumulative duration and magnitude of exposure. Persistent LDL-C elevation beginning in early adulthood may translate into substantial lifetime atherosclerotic burden even when short-term absolute risk remains low (15, 33, 40).

#### 7.4.4 Clinical implications

The lipid findings warrant confirmation under standardized conditions, verification of actual lipid-lowering therapy exposure and expected response, and evaluation for familial and secondary causes of dyslipidemia (33, 34, 40, 45–47). Lifestyle optimization remains fundamental, but its implementation should account for environmental influences, health literacy, socioeconomic circumstances, and the feasibility of sustained behavioral change (9, 10, 12, 21).

Figure 1 provides an educational histopathological illustration of established atherosclerotic plaque morphology and is independent of the clinical vignette. The images originate from the archival educational histopathology collection of the Department of Medical Diagnostics at Jagiellonian University Medical College, compiled over several years for teaching purposes from residual surgical specimens obtained through institutional clinical collaborations. They are fully anonymized, contain no patient identifiers or linked clinical, demographic, or outcome data, and cannot be traced to the individual described in this vignette. Accordingly, they represent archival educational material rather than patient-specific research and serve solely to illustrate the established morphological consequences of atherosclerotic disease discussed in this review (24, 40, 41, 44).

**Figure 1:**
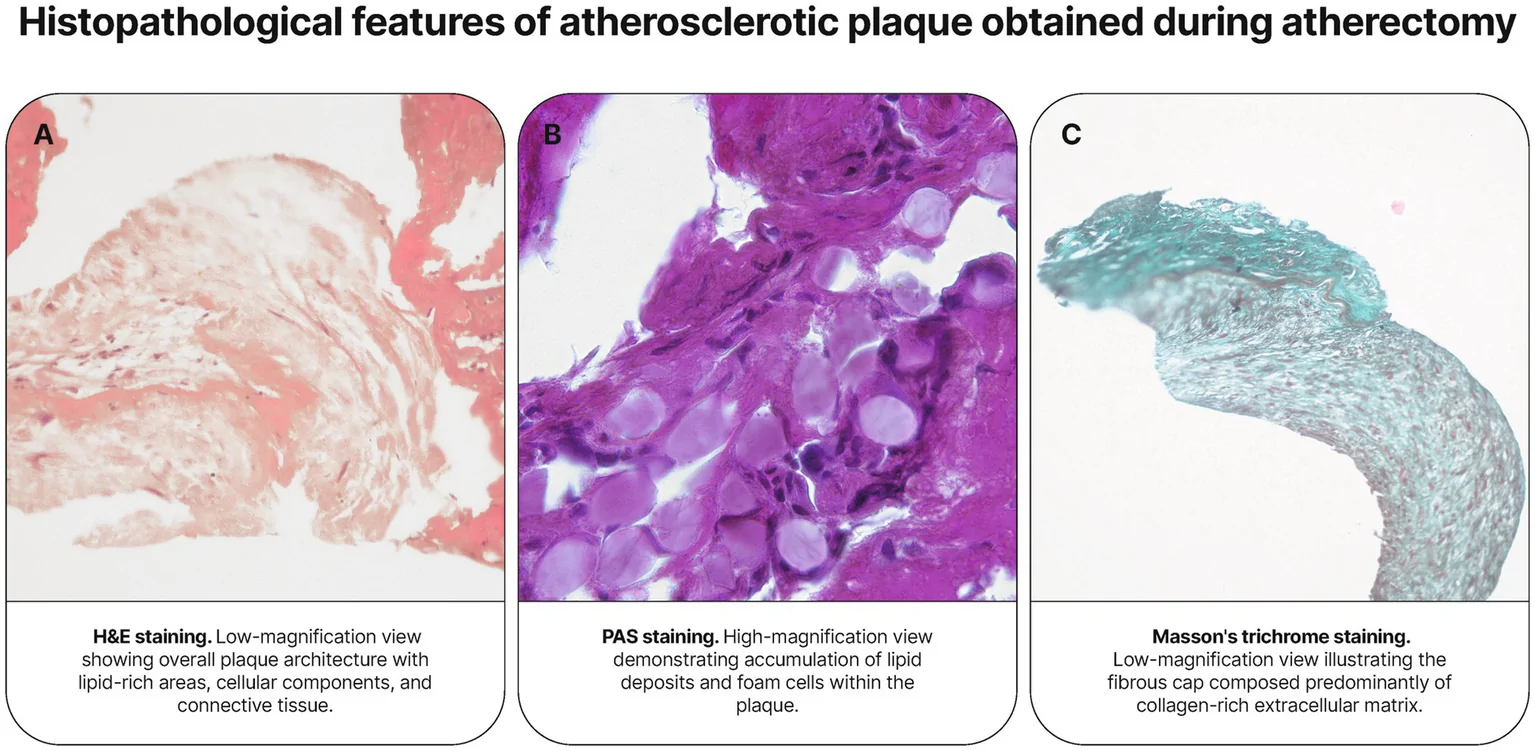
Representative histopathological features of advanced atherosclerotic plaque morphology in anonymized archival educational material. **(A)** Hematoxylin and eosin (H&E) staining demonstrating the overall architecture of an advanced atherosclerotic lesion, including marked intimal thickening, inflammatory cell infiltration, necrotic core formation, and arterial wall remodeling. **(B)** Periodic acid–Schiff (PAS) staining highlighting lipid-rich regions, macrophage-derived foam cells, and focal extracellular matrix alterations within the lesion. **(C)** Masson’s trichrome staining showing collagen deposition and fibrous cap organization, illustrating extracellular matrix remodeling and structural determinants of plaque stability. These representative histopathological findings illustrate established mechanisms of atherosclerotic plaque progression, including subendothelial lipid accumulation, chronic vascular inflammation, foam cell formation, extracellular matrix remodeling, and fibrous cap development (24, 40, 41, 44). The images originate from the archival educational histopathology collection of the Department of Medical Diagnostics at Jagiellonian University Medical College, compiled over several years for teaching purposes from residual surgical specimens obtained through institutional clinical collaborations. All images are fully anonymized and contain no patient identifiers, linked clinical information, demographic data, or outcome data. The material shown in this figure is entirely independent of the educational clinical vignette presented in this review and does not derive from the patient described therein. Accordingly, this figure represents anonymized archival educational material rather than patient-specific research, requiring no additional patient consent or institutional ethics committee approval. These histological images were obtained from an atherosclerotic plaque removed during atherectomy. The findings illustrate key morphological features of advanced atherosclerosis, including lipid accumulation, foam cell formation, and development of a fibrous cap. H&E, hematoxylin and eosin; PAS, periodic acid-Schiff; Masson, Masson's trichrome.

The broader educational message is that routinely available longitudinal data—including lipid trajectories, family history, treatment response, anthropometric changes, and metabolic context—can provide sufficient information to trigger timely preventive reassessment without requiring immediate recourse to increasingly complex biomarkers (8–10, 33).

### 7.5 Thyroid function as a potential secondary contributor requiring verification

#### 7.5.1 Interpretation of results in the clinical context

The patient’s thyroid-stimulating hormone (TSH) concentration was 3.742 μIU/mL, within the laboratory reference interval. Considered in isolation, this result does not indicate overt primary thyroid dysfunction. Nevertheless, because the patient had persistent dyslipidemia, gradual weight gain, an apparently limited response to lipid-lowering therapy, and a family history of thyroid disease, thyroid function remained relevant to the differential assessment of potential secondary contributors (46, 47, 54).

Importantly, a high-normal TSH value should not itself be pathologized. Although observational evidence has associated high-normal TSH concentrations with metabolic abnormalities in some populations (54), such associations do not establish thyroid dysfunction or demonstrate causality in an individual patient. Further endocrine evaluation should therefore depend on clinical pre-test probability, longitudinal findings, symptoms, examination, and repeat biochemical assessment rather than on a single value within the reference interval (46, 47).

#### 7.5.2 Thyroid function and lipid metabolism

Thyroid hormones contribute to cholesterol homeostasis through regulation of hepatic LDL receptor expression, cholesterol synthesis, bile acid metabolism, and pathways influencing clearance of ApoB-containing lipoproteins (46, 48). Clinically significant reductions in thyroid hormone activity can therefore impair LDL clearance and contribute to an atherogenic lipid phenotype (46–48).

Figure 2 summarizes the established mechanistic relationships linking thyroid dysfunction with altered lipoprotein metabolism, endothelial dysfunction, vascular inflammation, oxidative stress, and atherosclerosis. These mechanisms provide biological context for considering thyroid disease among potential secondary contributors to dyslipidemia, but they should not be interpreted as evidence that thyroid dysfunction is responsible for the lipid phenotype in this particular vignette (5, 46–48).

**Figure 2:**
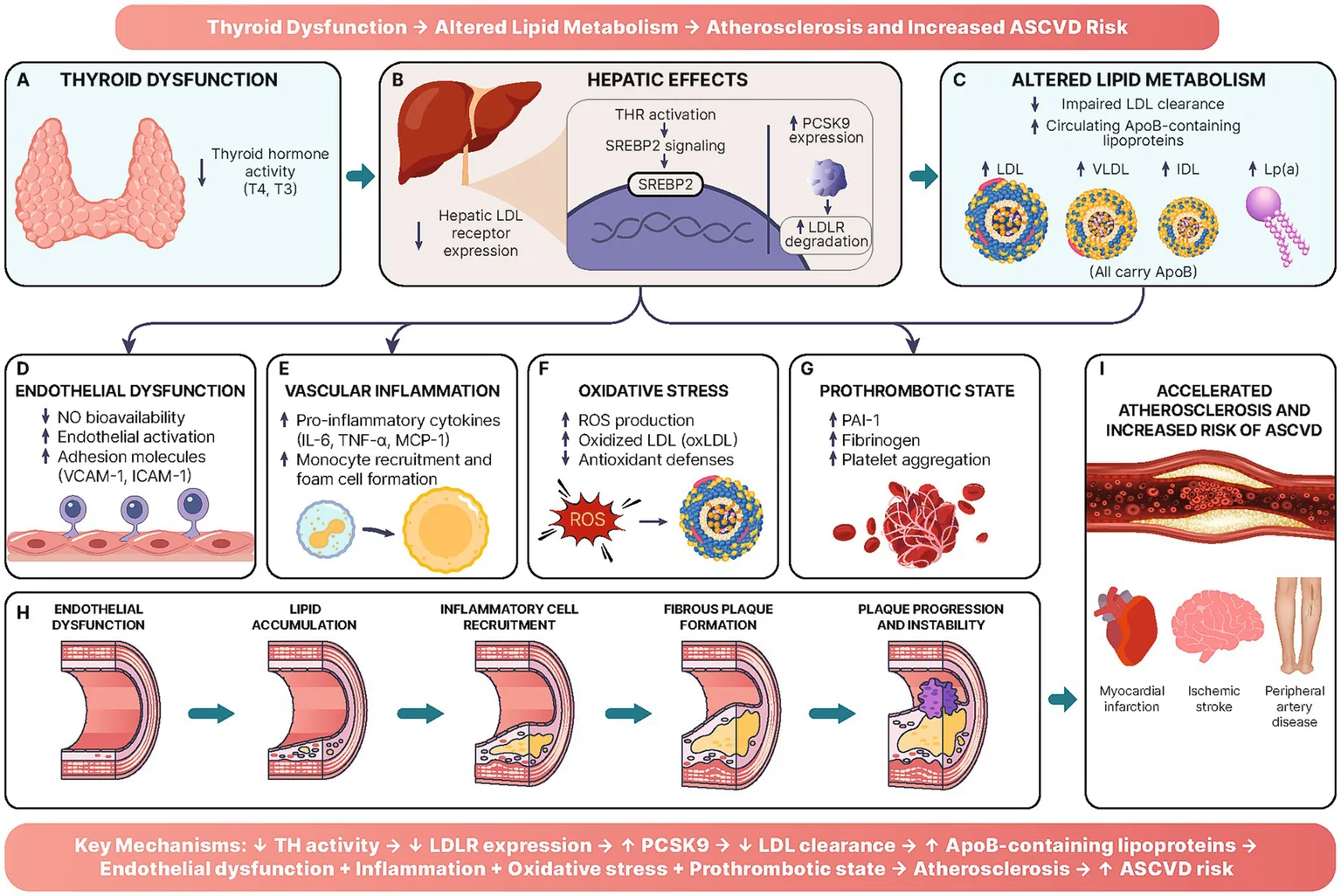
Mechanistic pathways linking thyroid dysfunction with altered lipid metabolism, vascular injury, and accelerated atherosclerosis. **(A)** Reduced thyroid hormone activity, particularly diminished triiodothyronine (T3) signaling, leading to impaired systemic metabolic regulation. **(B)** Hepatic effects of reduced thyroid hormone signaling, including decreased low-density lipoprotein receptor (LDLR) expression, dysregulation of sterol regulatory element-binding protein 2 (SREBP2) signaling and proprotein convertase subtilisin/kexin type 9 (PCSK9)-mediated LDLR regulation, resulting in impaired hepatic clearance of LDL particles. **(C)** Dysregulated lipoprotein metabolism characterized by impaired clearance and increased circulating concentrations of apolipoprotein B (ApoB)-containing lipoproteins, including low-density lipoprotein (LDL), very-low-density lipoprotein (VLDL), and intermediate-density lipoprotein (IDL), thereby promoting an atherogenic lipid profile. **(D)** Endothelial dysfunction associated with reduced nitric oxide (NO) bioavailability, endothelial activation, and increased expression of vascular and intercellular adhesion molecules, including VCAM-1 and ICAM-1. **(E)** Vascular inflammation characterized by increased pro-inflammatory signaling, including interleukin-6 (IL-6), tumor necrosis factor-α (TNF-α), and monocyte chemoattractant protein-1 (MCP-1), together with enhanced monocyte recruitment and foam cell formation within the arterial wall. **(F)** Oxidative stress resulting from increased generation of reactive oxygen species (ROS), oxidative modification of LDL particles (oxLDL), and impaired antioxidant defense mechanisms. **(G)** A prothrombotic milieu associated with alterations in fibrinolytic and coagulation pathways, including increased plasminogen activator inhibitor-1 (PAI-1) activity, elevated fibrinogen levels, and enhanced platelet activation. **(H)** Progressive atherosclerotic plaque development involving endothelial dysfunction, subendothelial lipid accumulation, inflammatory cell recruitment, foam cell formation, extracellular matrix remodeling, fibrous plaque formation, and, in advanced lesions, plaque instability. **(I)** Potential clinical consequences of accelerated atherosclerosis, including an increased risk of myocardial infarction, ischaemic stroke, and peripheral artery disease, thereby contributing to overall atherosclerotic cardiovascular disease (ASCVD) risk. TH, thyroid hormone; THR, thyroid hormone receptor; SREBP2, sterol regulatory element-binding protein 2; PCSK9, protein convertase subtilisin/kexin type 9; LDLR, LDL receptor; ApoB, apolipoprotein B; LDL, low-density lipoprotein; VLDL, very low-density lipoprotein; IDL, intermediate-density lipoprotein; Lp(a), lipoprotein(a); NO, nitric oxide; ROS, reactive oxygen species; PAI-1, plasminogen activator inhibitor-1; ASCVD, atherosclerotic cardiovascular disease.

#### 7.5.3 Interpreting an apparently limited response to lipid-lowering therapy

An unexpectedly small reduction in LDL-C should first prompt verification of treatment-related factors. Prescribed statin intensity, dosage, duration of therapy, adherence, tolerability, potential drug interactions, analytical variability, and the expected percentage LDL-C reduction should all be reviewed before a secondary endocrine mechanism is invoked (34, 45, 47).

Only after these factors have been adequately assessed should the evaluation expand toward plausible secondary biological contributors, including thyroid dysfunction, diabetes, nephrotic syndrome, selected hepatic disorders, and medication-related causes (34, 45–47). Familial hypercholesterolemia should be considered separately as a primary inherited dyslipidemia, particularly when marked LDL-C elevation is persistent from a young age or accompanied by a relevant family history (33, 34, 40).

#### 7.5.4 Proportionate thyroid evaluation

If thyroid disease remains clinically plausible, further investigation should be guided by pre-test probability. Repeat TSH measurement may be appropriate when the initial result is discordant with the clinical picture or when biochemical abnormalities evolve. Free thyroxine should be assessed when indicated by TSH results or sustained clinical suspicion, while thyroid autoantibody testing should be targeted to circumstances in which autoimmune thyroid disease is clinically plausible. Thyroid ultrasonography should primarily address structural indications rather than dyslipidemia alone (44–47).

This distinction is important because overt hypothyroidism has well-established effects on lipid metabolism and cardiovascular risk, whereas the clinical implications of mild or high-normal TSH abnormalities are considerably more heterogeneous (44, 46, 47, 54). The objective is therefore not to exclude thyroid involvement solely because TSH lies within the reference interval, nor to attribute dyslipidemia to thyroid dysfunction without confirmatory evidence.

The principal educational message is one of diagnostic proportionality: routine laboratory findings should progressively refine clinical probability and guide targeted investigation rather than be forced into a predetermined etiological explanation (33, 44–47).

### 7.6 The right specialist at the right stage of care

The integrated findings support a stepwise diagnostic pathway coordinated primarily within primary care rather than simultaneous referral to multiple subspecialists. Such an approach preserves care continuity, reduces redundant testing, and ensures that specialist consultation addresses a defined clinical question (9, 10).

Initial priorities include confirmation of the lipid and liver abnormalities under standardized conditions, verification of prescribed statin intensity and actual medication exposure, assessment of treatment tolerability, evaluation for primary and plausible secondary causes of dyslipidemia, and discussion of both short-term and lifetime cardiovascular risk with the patient (9, 33, 34). A predefined follow-up plan should specify responsibility for reviewing repeat results, monitoring therapeutic response, and initiating referral when appropriate.

Subspecialty consultation should therefore be targeted:

- Cardiology or lipid clinic: Appropriate for severe or persistent hypercholesterolemia, suspected familial hypercholesterolemia, uncertainty regarding treatment intensity or therapeutic targets, or failure to achieve an expected LDL-C response despite verified treatment exposure and adherence (33, 34, 40).
- Endocrinology: Appropriate when repeated biochemical findings, symptoms, physical examination, or family history sustain clinically meaningful suspicion of endocrine disease. A single TSH result within the reference interval does not, by itself, mandate specialist referral (44–47).
- Gastroenterology or hepatology: Appropriate when aminotransferase abnormalities persist or progress, when imaging indicates relevant hepatic pathology, or when the overall clinical profile suggests advanced MASLD or another chronic liver disorder. A single minimally elevated ALT concentration without corroborating evidence does not justify immediate specialist referral (6, 43, 53).

The effectiveness of this model depends less on the number of specialists involved than on the timing, purpose, and coordination of their involvement. Primary care should retain responsibility for longitudinal integration, while specialists address defined diagnostic or therapeutic questions and laboratory data are interpreted as trajectories rather than isolated results (8–10).

This approach embodies a fundamental principle of preventive cardiology: intervention is most valuable when cumulative risk is recognized and addressed before overt cardiovascular disease becomes clinically manifest (15, 33, 40, 49).

### 7.7 Educational synthesis: from isolated findings to an integrated prevention pathway

This vignette does not establish that a single occult disorder accounts for the patient’s lipid phenotype. Instead, it demonstrates how preventive opportunities may be missed when clinical and laboratory findings are interpreted episodically rather than longitudinally (8–10, 33). A BMI at the threshold of overweight, a minimally elevated ALT concentration, a family history of thyroid disease, a high-normal TSH value, or a reported limited response to statin therapy is individually insufficient to establish a specific diagnosis. Their combination, however, justifies a structured reassessment of persistent dyslipidemia, treatment exposure, potential primary and secondary etiologies, and cumulative lifetime cardiovascular risk (33, 34, 40, 43–47).

The vignette integrates four complementary dimensions of cardiometabolic prevention:

- Biological dimension: Persistent elevations in LDL-C and non-HDL-C represent prolonged exposure to ApoB-containing atherogenic lipoproteins and therefore have implications beyond a single laboratory measurement (24, 34, 40, 42, 43).
- Clinical dimension: Young age and absence of symptoms may create a misleading impression of low risk when short-term prediction is considered without cumulative lifetime exposure (27, 33, 40, 49).
- Diagnostic dimension: Laboratory findings require confirmation and contextual interpretation, including verification of actual medication exposure, consideration of familial dyslipidemia, assessment of metabolic liver abnormalities, and targeted evaluation of plausible secondary causes (33, 34, 43–47).
- Organizational dimension: Effective prevention depends on care continuity across primary care, laboratory medicine, clinical pharmacy, and selectively involved subspecialists rather than parallel investigations and disconnected referrals (8–10).

These dimensions can be translated into a practical, sequential prevention pathway:

1. Standardized confirmation (baseline verification): Confirm relevant lipid and biochemical abnormalities under appropriate standardized conditions and determine whether observed deviations are persistent (34, 43).
2. Pharmacological verification (adherence and exposure): Verify prescribed dosage, treatment intensity and duration, actual medication exposure, tolerability, and adherence before concluding that therapy has failed (34, 45).
3. Etiological assessment (primary and secondary causes): Investigate clinically plausible inherited and secondary contributors according to history, physical examination, laboratory phenotype, and pre-test probability (33, 34, 43–47).
4. Risk communication (patient-centered translation): Explain the distinction between short-term and lifetime cardiovascular risk in clear and clinically meaningful terms (9, 27, 33).
5. Shared goal setting (therapeutic agreement): Establish realistic lifestyle and pharmacological goals through shared decision-making, taking account of patient preferences, treatment burden, and feasibility (9, 33).
6. Targeted referral (subspecialty input): Refer selectively when confirmed abnormalities or unresolved clinical questions require specialist expertise (9, 33, 43–47).
7. Structured reassessment (longitudinal follow-up): Define responsibility and timing for reassessment of laboratory trajectories, treatment response, therapeutic targets, and emerging risk factors (8, 9, 33).
8. Feedback and continuity (PROMs and PREMs): Incorporate patient-reported outcomes and experiences to identify barriers related to health literacy, treatment burden, access, adherence, and coordination of care (9, 12, 14).

The central lesson is that increasing the volume of diagnostic testing does not inherently improve preventive care. Effective prevention requires identification of findings that are reproducible, clinically coherent, and actionable, while avoiding both underdiagnosis and unnecessary medicalization (9, 10, 33).

The evidence-to-practice gap may therefore persist even when individual diagnostic tests, prescriptions, and specialist consultations are appropriate in isolation. Prevention becomes ineffective when responsibility for integrating these elements into a coherent longitudinal strategy is unclear (8–10). Routine laboratory data acquire their greatest clinical value when interpreted within the patient’s broader health trajectory, communicated effectively, and translated into proportionate and timely action (9, 10, 33).

Accordingly, this vignette serves as a translational bridge between the evidence reviewed in the preceding sections and routine clinical practice. It demonstrates how biological, clinical, diagnostic, behavioral, and organizational dimensions intersect in the prevention of cardiometabolic disease and reinforces the central premise of this review: preventive knowledge produces meaningful clinical benefit only when health systems can recognize actionable patterns early and convert them into coordinated, sustained, and patient-centered care (8, 9, 33).

## 8 Summary and conclusions

This review integrates epidemiological, pathophysiological, clinical, organizational, economic, and implementation evidence to explain why major advances in cardiometabolic prevention have not translated into commensurate improvements in population health. The central limitation is no longer primarily a lack of scientific knowledge or effective therapies, but the persistent failure to translate established evidence into timely, coordinated, equitable, and sustained clinical action. Delayed risk recognition, therapeutic inertia, fragmented care pathways, insufficient support for long-term adherence, health inequities, and incomplete integration of clinical, laboratory, behavioral, and patient-reported information collectively sustain this evidence-to-practice gap (1, 4, 8–12, 33).

Cardiometabolic prevention must therefore move beyond episodic, disease-specific management toward a lifelong, integrated model of care. Effective prevention requires early recognition of cumulative biological risk, proportionate intervention based on short-term and lifetime risk, coordinated longitudinal care, sustained patient engagement, and health-system accountability for monitoring outcomes and closing gaps across the preventive pathway (9, 10, 14, 33, 40, 49). Multidisciplinary involvement is essential, but its value depends on coordination rather than parallel specialist activity: primary care should maintain longitudinal oversight, laboratory medicine should support integrated interpretation of biological trajectories, and subspecialists should address clearly defined diagnostic or therapeutic questions (9, 10, 14, 33).

The principal implication is that preventive success should no longer be judged solely by the availability of increasingly sophisticated risk models, biomarkers, or therapies. Their clinical value depends on whether they lead to earlier recognition, appropriate treatment initiation and intensification, sustained adherence, meaningful patient participation, equitable access, and measurable improvement in long-term outcomes. Patient-reported outcome measures (PROMs), patient-reported experience measures (PREMs), digital tools, implementation science, and health-economic evaluation should therefore complement conventional biomedical endpoints when assessing the quality and sustainability of preventive care (8–12, 14).

Ultimately, bridging the evidence-to-practice gap requires cardiometabolic prevention to become a shared, system-wide responsibility extending across the life course. When biological evidence, clinical reasoning, coordinated care, patient engagement, and health-system accountability operate as a single preventive continuum, prevention can move from isolated recommendations and episodic encounters toward sustained improvements in cardiometabolic health and patient-centered outcomes (8–12, 14, 33).
